# Cheminformatic Profiling and Hit Prioritization of Natural Products with Activities against Methicillin-Resistant *Staphylococcus aureus* (MRSA)

**DOI:** 10.3390/molecules26123674

**Published:** 2021-06-16

**Authors:** Samson O. Oselusi, Samuel A. Egieyeh, Alan Christoffels

**Affiliations:** 1 School of Pharmacy, University of the Western Cape, Bellville, Cape Town 7535, South Africa; 3866891@myuwc.ac.za; 2 South African Medical Research Council Bioinformatics Unit, South African National Bioinformatics Institute, University of the Western Cape, Cape Town 7535, South Africa; alan@sanbi.ac.za

**Keywords:** cheminformatics, profiling, natural products, methicillin-resistant *Staphylococcus aureus* (MRSA), hit prioritization, hit-to-lead optimization, drug-likeness, desirability score

## Abstract

Several natural products (NPs) have displayed varying in vitro activities against methicillin-resistant *Staphylococcus aureus* (MRSA). However, few of these compounds have not been developed into potential antimicrobial drug candidates. This may be due to the high cost and tedious and time-consuming process of conducting the necessary preclinical tests on these compounds. In this study, cheminformatic profiling was performed on 111 anti-MRSA NPs (AMNPs), using a few orally administered conventional drugs for MRSA (CDs) as reference, to identify compounds with prospects to become drug candidates. This was followed by prioritizing these hits and identifying the liabilities among the AMNPs for possible optimization. Cheminformatic profiling revealed that most of the AMNPs were within the required drug-like region of the investigated properties. For example, more than 76% of the AMNPs showed compliance with the Lipinski, Veber, and Egan predictive rules for oral absorption and permeability. About 34% of the AMNPs showed the prospect to penetrate the blood–brain barrier (BBB), an advantage over the CDs, which are generally non-permeant of BBB. The analysis of toxicity revealed that 59% of the AMNPs might have negligible or no toxicity risks. Structure–activity relationship (SAR) analysis revealed chemical groups that may be determinants of the reported bioactivity of the compounds. A hit prioritization strategy using a novel “desirability scoring function” was able to identify AMNPs with the desired drug-likeness. Hit optimization strategies implemented on AMNPs with poor desirability scores led to the design of two compounds with improved desirability scores.

## 1. Introduction

The incidence of bacterial resistance to antibiotics is growing at an alarming rate across the globe. It is one of the major causes of morbidity, mortality, and economic burden [1,2,3]. In the United States, about 2 million patients are infected with these bacteria, and more than 23,000 cases of death are annually attributed to infections that they cause [1,2]. Continuous and inappropriate uses of antibiotics, including the transfer of resistance within and between unrelated species, are some of the major factors responsible for developing antibiotic resistance [4,5]. A more serious concern the failure of pharmaceutical industries to develop new antibiotics because of poor economic returns and regulatory obstacles [2]. All these have contributed to the growing rate of resistance among pathogenic organisms.

Methicillin-resistant *Staphylococcus aureus* (MRSA) is one of the commonly known antibiotic-resistant bacteria and life-threatening pathogens. MRSA has developed resistance to methicillin and other β-lactam drugs such as amoxicillin, cephalosporins, oxacillin, penicillin, and tetracycline, which were formally used in its treatment [6]. This superbug has now left fewer treatment options available, thereby making it more challenging to control. Recent studies have identified MRSA as a drug-resistant pathogen of international concern, requiring urgent research to discover and develop new and potent antimicrobial agents [7].

The various therapeutic advantages of compounds sourced from nature have been reviewed [8,9,10,11]. In addition, about 80% of antibiotics that are recently approved for treating many life-threatening infections are sourced from natural products (NPs) [10]. This success has been linked to the considerable bioactive or chemical spaces and broad diversities of NPs, giving them an edge of uncovering distinct structural groups over those obtained from synthetic antibiotics [12]. Therefore, it is anticipated that NPs might win the fight against MRSA.

Researchers have reported the in vitro activities of NPs against multiple-drug-resistant bacteria, including MRSA [13,14,15]. However, many of these compounds have not made their way into drug development pipelines [16]. This lack of progress can be attributed to more than a decade and/or a cost of about USD 2.5 billion required to transform NPs from hit compounds to drug candidates. These difficulties are connected to attempts to balance efficacy and safety deficiencies and properties related to absorption, distribution, metabolism, excretion, and toxicity (ADMET), which are crucial for the success of any drug candidate [16,17,18,19]. Therefore, assessment of drug-likeness based on some key physicochemical properties (PP) [19,20,21,22] is crucial at the early stage of drug discovery development (17). A wide range of computational techniques can be used in modern drug discovery projects to predict the drug-likeness of hit compounds [23]. Taking advantage of these tools, the present study conducted cheminformatic analysis and data mining towards hit profiling, hit prioritization, and hit optimization of AMNPs. The results revealed that most of the AMNPs have the desired drug-like properties, and those with undesired properties may be optimized to improve the desired properties. The process implemented in this study could guide drug developers to realize the full prospects of available data on AMNPs in the discovery of new antibiotics.

## 2. Results and Discussion

This study was set out to profile anti-MRSA NPs (AMNPs) for drug-likeness and to identify their potential for “hit- to-“lead” optimization. The datasets (reportedly sourced from plants, microbes, and marine organisms) consist of 111 AMNPs with their bioactivity obtained from a recent literature search (Table S1, in the Supplementary Material). The reported bioactivity of these compounds was normalized (Table S1) and grouped into three classes: significantly active (SA), moderately active (MA), and negligibly active (NA). More details of the procedures for data collection and preparation can be found in the method section. Overall, the AMNPs were 45.9% SA, 40.5% MA, and 13.5% NA. The results and implications of the findings from this study are provided below.

### 2.1. Molecular Descriptors and Physicochemical Properties of AMNPs and CDs

The key molecular descriptors and physicochemical properties of the overall AMNPs and the different categories (SA, MA, and NA) were profiled for drug-likeness using currently prescribed oral drugs for MRSA (CDs) (Figure S1, in the Supplementary Material) as reference. Here we present the distribution of these properties and their implications.

#### 2.1.1. Molecular Weight

**Figure 1:**
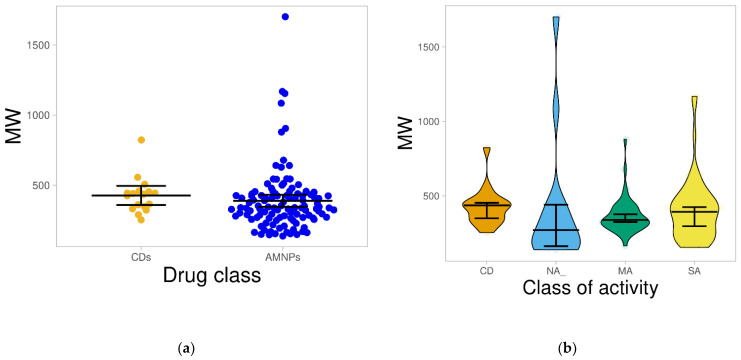
Distribution of the molecular weight of the datasets. (**a**) presents the distribution of AMNPs and CDs on a plot. The line that divides the plots denotes the mean of the distribution, which shows no statistical difference (*p* > 0.05) between the CDs and AMNPs. (**b**) shows the violin plot of the CDs compared with the various categories of the AMNPs. The boxes indicate the distribution of each of the datasets; the line that divides each of the boxes represents the median of the distributions. The black bars in both figures indicate a 95% confidence interval.

Molecular weight (MW) is one of the key parameters required for oral bioavailability [22]. Compounds with MW above 500 Dalton have been suggested to have a higher tendency for absorption problems [20,21]. The results from this study depict that about 87% and 88% of the AMNPs and CDs have MW less than 500 Dalton (Figure 1a). The mean of the AMNPs (389.3 Dalton) was not statistically different from that of the CDs (427.2 Dalton), suggesting that most of the AMNPs will be bioavailable via the oral route.

Additionally, most of the AMNPs could have “room” for the addition of required bioisosteres towards improving specific drug-like properties during the “hit”-to-“lead” optimization process. The few complex compounds in the CDs, mainly sourced from fungus, may explain the higher MW observed in the CDs. The result for the distribution of MW across the AMNP categories is presented in Figure 1b, where the median MW of SA (392.49) is highest and closest to the CDs (435.88). However, the largest fraction (19.6%) of heavy compounds (MW > 500 Da) was found among the SA groups in comparison with other AMNPs. Furthermore, most of the compounds within the various categories of the AMNPs were distributed below MW of 500 Da, and their mean MW, 366.47, 401.82, and 405.75 for MA, NA, and SA, respectively, were not significantly different (*p* > 0.05) from that of the CDs (427.17) (Figure 1b). High MW has been associated with more significant bioactivity because of the propensity/tendency of big compounds to encumber the binding pockets of drug targets to bring about bioactivity [21,24]. This might explain why the AMNPs with higher MW showed greater bioactivity.

#### 2.1.2. Calculated Octanol/Water Partition Coefficient (cLogP)

**Figure 2:**
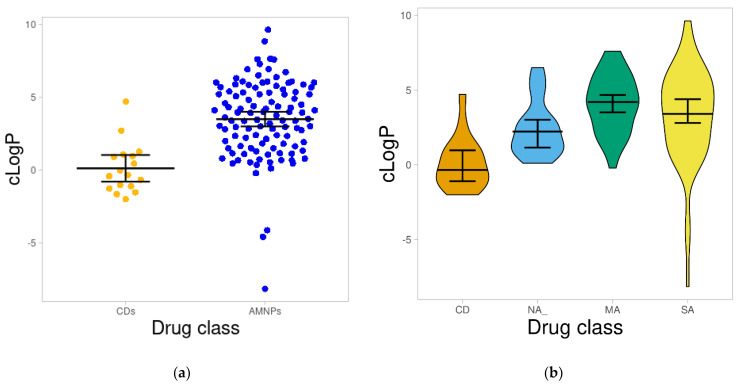
Distribution of cLogP for the datasets. (**a**) is a swarm plot showing the cLogP of AMNPs and CDs. (**b**) shows a violin plot of the various categories of AMNPs and CDs where the boxes indicate the distribution of each of the datasets; the line that divides the boxes represents the median of the distributions. The average cLogP for all the categories of the AMNPs is significantly higher (*p* < 0.05) than that of the CDs. The black bars in both figures indicate a 95% confidence interval.

The cLogP is used to estimate the hydrophilicity of a compound [25]. This descriptor also contributes to drug-receptor interaction and the solubility and absorption of bioactive compounds. Figure 2a illustrates the predicted distribution of cLogP for AMNPs compared with the CDs.

Compounds with cLogP values above 5 are not likely to be well absorbed. This is because high LogP tends to compromise the bioavailability of a bioactive molecule [26]. In this study, the means of both AMNPS and CDs were lower than 5, and about 68% of the AMNPs revealed cLogP values below 5 (Figure 2a). In addition, CDs had cLogP values below 5, and about 60% of these compounds showed negative cLogP (Figure 2a). As shown in Figure 2b, most of the compounds within each of the categories of AMNPs had cLogP below 5, and the average mean values (NA = 2.45, MA = 4.1, SA = 3.27) were considerably different (*p* < 0.05) from that of the reference compound (CDs = 0.12). This can be an indication that CDs are more hydrophilic and with poor membrane permeability compared with AMNPs.

On the other hand, more positive cLogP values observed for AMNPs might indicate that they are more hydrophobic than most CDs. The more hydrophobic a molecule is, the more likely it is to bind to a target, resulting in greater bioactivity [26,27]. Therefore, the correlation between the cLogP values of AMNPs and the reported bioactivity (particularly those with MIC ≥ 1) was explored. Surprisingly, there was a weak negative correlation (−0.074) between cLogP and the bioactivity of AMNPs (Figure S2, in the Supplementary Material). This suggests that other molecular descriptors may have contributed to the observed differences in the bioactivities of AMNPs.

#### 2.1.3. Hydrogen Bond Acceptors and Donors

**Figure 3:**
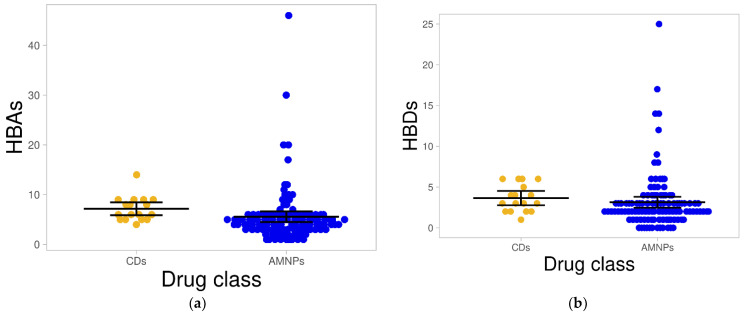
Distribution of hydrogen bonds for both AMNPs and CDs. (**a**,**b**) presents swarm plots showing the distribution of HBAs and HBDs, respectively, for both datasets. The black bars in both figures show a 95% confidence interval in the distribution.

The hydrogen bond is a crucial property in drug–receptor interaction that may lead to pharmacological action. It also plays a vital role in membrane transport and drug distribution in a biological system [28]. In this study, the most obvious observation regarding the hydrogen bond was an accumulation of both CDs and AMNPs with few numbers: ≤10 for hydrogen bond acceptors (HBAs) and ≤5 for hydrogen bond donors (HBDs). However, the average hydrogen bonds for AMNPs (HBAs = 5.57 and HBDs = 3.14) were generally lower (*p* < 0.05) than those for CDs (HBAs = 7.18 and HBDs = 3.65) (Figure 3a,b).

**Figure 4:**
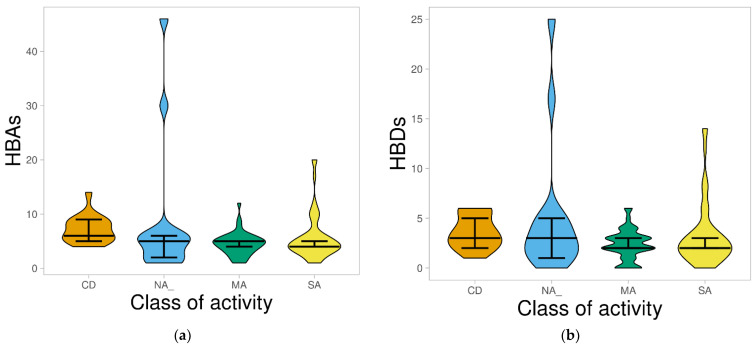
Violin plots of HBAs (**a**) and HBDs (**b**) against the class of activity. The plots show the distribution of CDs compared with the various categories of AMNPs, where the boxes indicate each of the datasets; the line that divides the boxes represents the median of the distributions. The black bars in both figures show a 95% confidence interval.

The distribution of HBAs and HBDs for the different categories of AMNPs was also evaluated and compared with that of CDs. Figure 4a shows that for HBAs, a median of 5 for both NA and MA among AMNPs was highest and closest to the reference (CDs = 6). Similarly, Figure 4b depicts that NA had the highest and the same median [3] as the CD. However, the distribution of both properties among the different categories reflects that their average values were generally moderate (HBAs: CD = 7.18, MA = 4.5, NA = 8.47, SA = 5.65; and HBDs: CD = 3.65, MA = 2.33, NA = 5.07, SA = 3.27), as they were all below the required threshold of 10 and 5 for HBAs and HBDs, respectively. The lower average values of AMNPs (particularly the SA and MA) might suggest that they have space for structural modification during the “hit”-to-“lead” and lead optimization phase.

#### 2.1.4. Total Polar Surface Area (TPSA)

**Figure 5:**
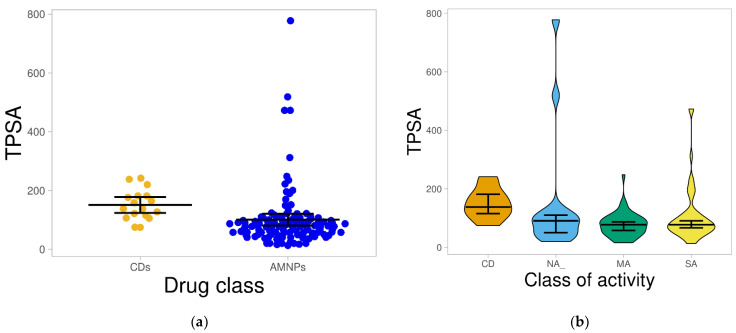
Distribution of TPSA for the datasets. (**a**) shows the distribution of TPSA for AMNPs compared with that for CDs; the average value for AMNPs was considerably lower (*p* < 0.05) than that for CDs. (**b**) shows the distribution of the various categories of AMNPs compared with that of CDs. The boxes indicate the distribution of each dataset; the line that divides the boxes shows the median of the distributions. The black bars in both figures indicate a 95% confidence interval.

Studies have shown that molecules with TPSA above 140 Å^2^ are not likely to penetrate through the intestinal membrane [29,30]. The results from this study depict that approximately 88% of AMNPs as against 53% of CDs had TPSA below 140 Å^2^. The mean of the distribution also shows that both datasets were statistically different (*p* < 0.05) from each other (Figure 5a).

The TPSA was significantly higher (*p* < 0.05) in CDs than MA and SA categories. Therefore, it can be inferred that most of the AMNPs (especially the MA and SA categories) might have prospects for better intestinal epithelial permeability than the CDs, and that they may be pursued in the development of anti-MRSA drug candidates. The higher TPSA values observed for the CDs may relate to their large MW because there is a positive correlation (r = 0.679) between these two parameters (MW and TPSA) (Figure S3, in the Supplementary Material).

#### 2.1.5. Rotatable Bond (RTB) Count

**Figure 6:**
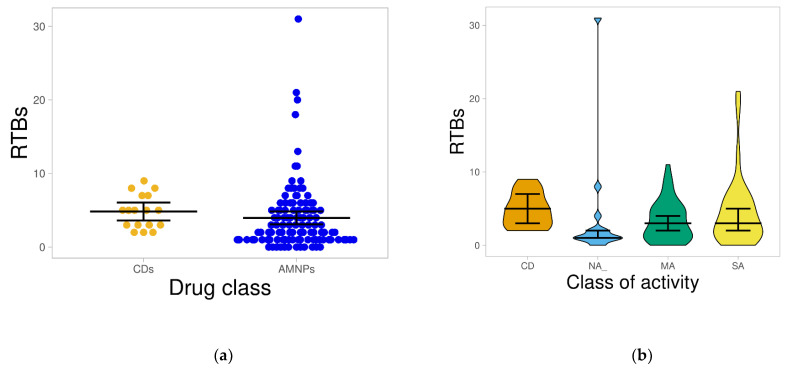
Distribution of rotatable bonds for the datasets. (**a**) shows the plot of RTBs for AMNPs compared with that for CDs, where both classes showed comparable distribution. (**b**) shows the distribution of the various categories of AMNPs compared with that of CDs. The boxes depict each of the datasets, while the line that divides the boxes represents the median of the distributions. The black bars in both figures indicate a 95% confidence interval.

The number of RTBs has a direct effect on the flexibility of a molecule. It is employed to predict how compounds transverse the membrane. Therefore, it is used to determine the bioavailability of compounds via the oral route and gastrointestinal absorption [31,32]. Previous studies have established an RTB value of not more than 10 as one of the selection criteria for determining the oral bioavailability of drug candidates [22,32]. Our findings reveal that all of the CDs and approximately 94% of the AMNPs had RTBs not more than 10, and the average value for both datasets (CDs = 4.82, AMNPs = 3.96) was not statistically different (*p* > 0.05) from each other (Figure 6a).

As illustrated in Figure 6b, most of the compounds within each of the three categories of AMNPs were distributed below RTBs of 10. However, the median RTBs for both MA and SA [3] were the closest and most comparable to CDs [5]. The average distribution of SA (4.59) also revealed the highest and most comparable AMNPs to the CDs, which showed an average RTB of 4.82. Therefore, most of the AMNPs might have higher chances of good oral absorption.

### 2.2. Profiling Drug-Likeness of AMNPs

#### 2.2.1. Prediction of Absorption and Distribution

Some predictive rules have been developed to efficiently predict the oral absorption of a molecule [33]. These rules provide guidelines for the early identification of compounds with an increased chance of high oral absorption. In this study, the predictive rules of Lipinski, Veber, and Egan were used to investigate the consistency of drug-likeness among AMNPs with known anti-MRSA drugs (CDs).

**Figure 7:**
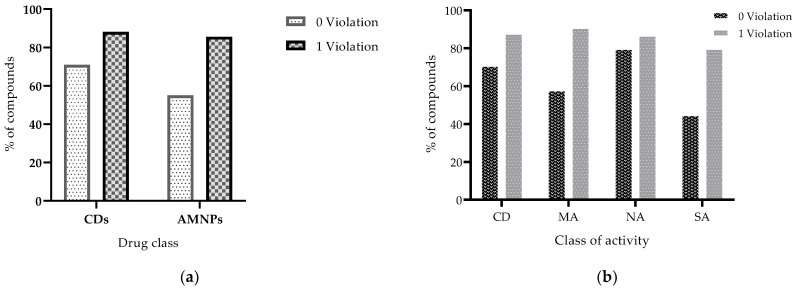
Histograms of the rule-of-five compliance based on the principle that drug-like compounds could break ≤ 1 of the rules. (**a**) shows that up to 71% and 55% of CDs and AMNPs, respectively, comply without any violation, while 85% of AMNPs violate not more than 1 of the rules. (**b**) shows that up to 88% of the CDs break not more than 1 of Lipinski’s rule, and among the AMNP categories, 45%, 58%, and 80% of SA, MA, and NA, respectively, obey the rule of five.

Lipinski’s rule describes molecules with MW < 500, cLogP < 5, HBDs < 5, and HBAs < 10 as more likely to have prospects for good oral absorption and permeation [21,32]. Figure 7 displays the results for compliance of the datasets with this rule. Overall, approximately 55% and 71% of AMNPs and CDs, respectively, passed Lipinski’s rule without any violation (Figure 7a). Similarly, among the AMNP categories, 45%, 58%, and 80% of SA, MA, and NA, respectively, obeyed the rule of five without any violation (Figure 7b).

Lipinski mentioned that a compound that violates ≤ 1 of the rules can still be considered drug-like [32]. We set out to check the proportion of AMNPs that adhered to Lipinski’s rule after violating not more than 1 of the parameters within the rule. Table 1 shows the proportion of the compounds that would be left when MW was violated, and the rest of the parameters were left alone, when cLogP was violated and the rest of the parameters were within the rule, when HBAs were violated and the other properties obeyed the rule, when HBDs were violated and the other three parameters complied with the rule. Overall, up to 85% and 88% of AMNPs and CDs, respectively, obeyed Lipinski’s rule with not more than one violation.

**Figure 8:**
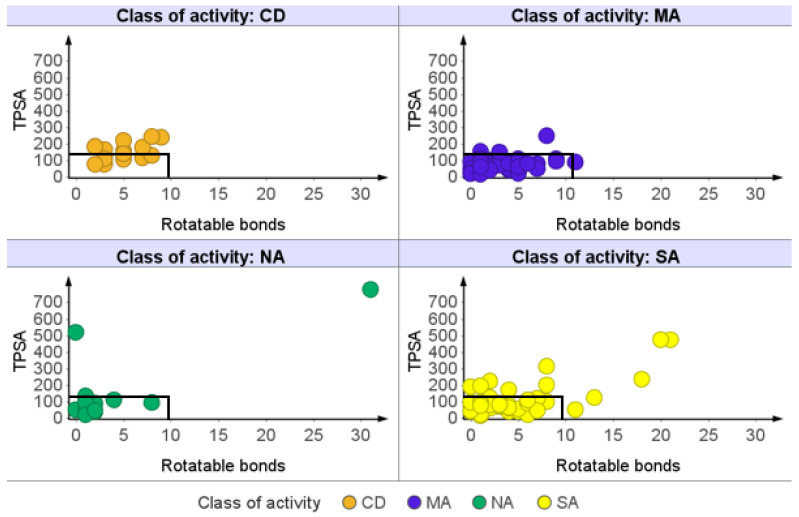
Prediction of oral absorption based on Veber’s model. Each of the panels represents a class of activity (CDs and subgroups of AMNPs). More than 78% of each of the categories of AMNPs and 53% of those of CDs (markers within the highlighted parts) obeyed Veber’s rule without any violation.

Among the categories of AMNPs, almost all (80% to 87%) of the NA molecules were found within the space of this rule (Table 1). This might indicate that the less active category of AMNPs (NA) has the potential to become orally active drugs. In another way, this observation reinforces the idea that bioactivity does not guarantee that a compound will be useful because drug-likeness is needed to determine the efficacy and selectivity of such compound. Furthermore, the lower compliance observed among SA and MA might suggest that there might be a need to optimize more for the physicochemical properties of the active compounds (MA and SA categories) to balance their potency with efficiency. The result obtained for oral absorption based on Veber’s rule (RTB ≤ 10 and TPSA ≤ 140 Å^2^) is presented in Figure 8.

Overall, 86% of AMNPs and 53% of CDs were within the space of the rule. For the AMNP categories, all compounds in NA, except tannic acid (TPSA = 777.98 and RTB = 31) and punicalagin (TPSA = 518.76 and RTB = 0), were in compliance with Veber’s rule. Additionally, more than 78% of both MA and SA categories obeyed the rule. Hence, there are greater chances for most of the AMNPs to achieve permeability at Veber’s limits than most of the currently prescribed anti-MRSA drugs.

**Figure 9:**
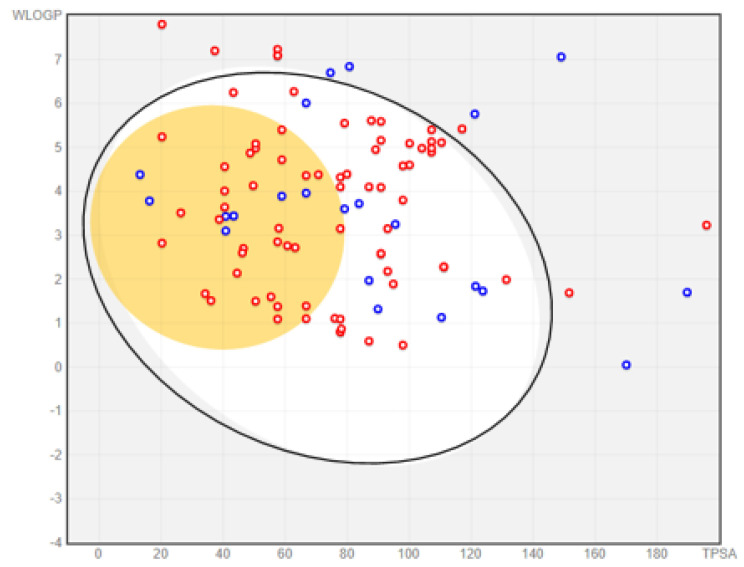
BOILED-Egg predictive model for absorption and bioavailability of AMNPs. The markers (dots representing AMNPs) within the white region are occupied by molecules (77% of AMNPs) that are most likely to be passively absorbed by the gastrointestinal tract (HIA). Those markers within the yellow region show AMNPs (34%) that are most likely to pass through the BBB. The markers that are outside the white region and few others that are out of the range (WlogP > 8 and TPSA > 200) are compounds that might be poorly absorbed. The blue and red markers represent substrates and non-substrates of p-gp, respectively.

Human intestinal absorption (HIA), permeation through the blood–brain barrier (BBB), and drug assessment for substrates of p-glycoproteins (p-gp) are crucial pharmacokinetic properties. They can be used in the early discovery process to determine the extent of intestinal absorption of a bioactive compound in humans [33,34,35,36]. The result presented in Figure 9 reveal that more than 77% of the AMNPs (compounds found within the white and yellow regions) were likely to be passively absorbed.

The result also shows that 76% (red markers) of the AMNPs that showed the prospect for HIA permeant might be non-p-gp substrates (i.e., not efflux from cells). Conversely, for the reference data (CDs), about 41% were predicted to have high HIA; among these compounds, 43% were non-p-gp substrates (Figure S4, in the Supplementary Material). One of the crucial screenings during the early stage of the drug discovery process is to check whether the biologically active molecules are substrates of p-gp. This is because p-gp functions to decrease cellular uptake, absorption, oral bioavailability, distribution, and retention time of drugs in the body through a unidirectional lipid flippase pathway [37]. The p-gp can limit the effective concentration of bioactive molecules at the desired cellular sites, leading to the rapid development of resistance, especially for anti-infective compounds. Therefore, more than 76% AMNPs that were predicted as non-substrates of p-gp in this study could have prospects for good oral absorption and bioavailability. For the reference compounds, sulfamethoxazole and chloramphenicol are a few examples of anti-MRSA drugs that are known for their good absorption and bioavailability as contained in the drug bank (www.drugbank.ca). On the other hand, omadacycline and doxycycline, among others, are also known for their poor oral absorption (www.drugbank.ca). Therefore, the present study is consistent with the established pharmacokinetic profile of CDs.

Furthermore, it was observed that approximately 34% of the AMNPs (i.e., 38 out of the total compounds) that showed a prospect for HIA (AMNPs found within the yellow region) could permeate the BBB, while none of the CDs showed a propensity for permeation of this barrier (Figure S4). The poor BBB permeability of the CDs could be because of their negative average cLogP values. The BBB is a major hindrance in the development of drugs for the central nervous system (CNS) [38]. This has made CNS infections that are caused by multi-drug-resistant organisms, such as multi-drug-resistant Gram-negative aerobic bacilli, MRSA, penicillin-resistant pneumococci, and other organisms, continually result in serious health threats [37,38]. Therefore, the AMNPs with predicted BBB in this study might be desirable for these CNS infections.

#### 2.2.2. Predicted Metabolism of AMNPs and Identification of Their Metabolites

Orally administered drugs are prone to extensive biotransformation in the liver such that their bioavailability and efficacy are extremely reduced [39]. Therefore, the propensity for the metabolism of AMNPs by phase 1 and phase 2 enzymes was evaluated. The results summarized in Table 2 reveal that 59% and 71% of AMNPs are likely to be metabolized by phase 1 and 2 enzymes, respectively, while more than 50% of AMNPs may be metabolized by both phase 1 and phase 2 enzymes.

**Figure 10:**
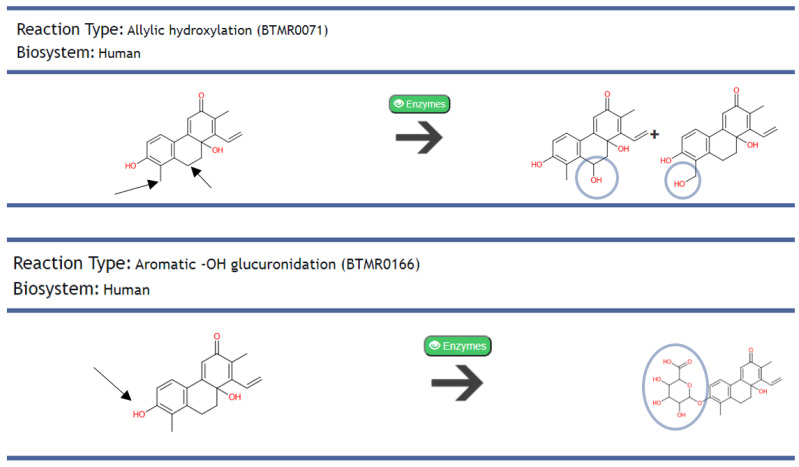
Showing HTML documents containing the results for biotransformation prediction of an AMNP (juncuenin D). The first line illustrates the structure of the parent compound (left side of the reaction), the enzymes (Cytochrome P450) that may likely act on it, and the products of the reactions (right side of the reaction) during phase 1 metabolism. The second line illustrates the parent structure (left side of the reaction), the enzymes (UDP-glucuronosyltransferase) that may likely act on it, and the predicted metabolite (right side of the reaction) during phase 2. Each of the circles represents the points of transformation, while the arrow points to the atom that is transformed.

A total of 20% of the AMNPs are not likely to be transformed by phase 1 and phase 2 enzymes. The obtained result for phase 1 reveals that 60% of the AMNPs may suffer the first-pass biotransformation while passing through the liver. This could consequently reduce the bioavailability of these compounds before reaching their targets. In contrast, the result for phase 2 shows that 71% of the AMNPs may produce metabolites at this stage, implying that most of the AMNPs could be readily excreted out of the body. The structural representation of the biotransformation result for one of the AMNPs (juncuenin D) is presented in Figure 10 below.

Juncuenin D was metabolized to produce two and one metabolites in phase 1 and phase 2, respectively. It has been reported that metabolites can cause adverse effects or become active products in the pharmacology of the parent molecules [40]. Therefore, biologically active categories of AMNPs (SA and MA) with pharmacologically active metabolites might prospect for prolonged action of these compounds in the body. Similarly, active metabolites formed by less active AMNPs (NA) may be considered further for advancement rather than the parent compounds. Hence, recognizing the pharmacological prospects of the AMNPs metabolite can be necessary to prevent their efficacy from being compromised at a later stage of drug development [39,40].

#### 2.2.3. Predicted CYP450 Inhibitory Potential of AMNPs

**Figure 11:**
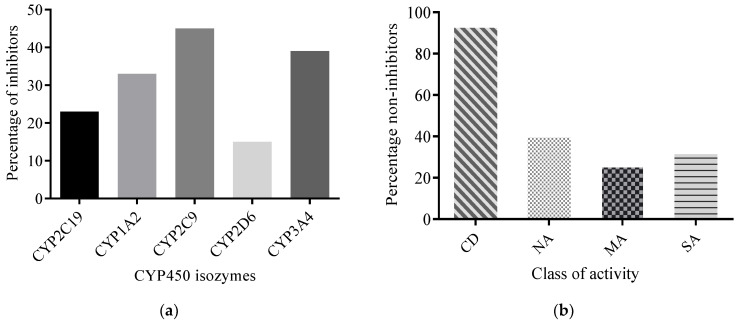
Percentage of AMNPs that are CYP450 isozyme inhibitors. (**a**) shows the percentage of AMNPs that are inhibitors of one or more CYP450 isozymes. (**b**) shows the percentage of the CD and AMNP categories that are non-inhibitors of CYP450 isozymes.

Potent inhibitors of CYP3A4 isozymes are not desirable in drug discovery as they may result in drug–drug interactions [41]. The results from this study reveal that between 15% and 45% of the AMNPs returned “YES” for inhibition of one or more of the isozymes (Figure 11a). A closer look at the AMNPs reveals that about 36 compounds (representing 41%, 27%, and 33% of NA, MA, and SA, respectively) might not inhibit any of the isoforms (Figure 11b).

Similarly, of all reference compounds (i.e., CDs) except DB225, cloxacillin was predicted as a non-inhibitor of CYP450 isoforms. Inhibition of CYP450 isoforms has led to the market withdrawal of many drugs, causing loss of valuable time and resources and drug lag [42]. Therefore, to avoid drug–drug interactions with current drugs and prevent the colossal waste that may come from the withdrawal of drug candidates at a later stage, in silico prediction of CYP enzyme inhibition for the AMNPs at the early stage of drug discovery is desirable. The AMNPs predicted to inhibit CYP450 enzymes in this study should be given less consideration during hit selection irrespective of their bioactivity. This is because of their potential to interact with other drugs.

#### 2.2.4. Toxicity Profiling of AMNPs

The prediction of the toxicity of drug candidates is one of the essential components of modern drug discovery. In this study, the toxicity of AMNPs was predicted by Osiris DataWarrior software. The result reveals that about 58.6% and 53.9% of AMNPs and CDs, respectively, may likely have negligible or no toxicity effects (Figure S5, in the Supplementary Material). For the categories of AMNPs, at least 47%, 56%, and 59% of NA, MA, and SA, respectively, returned “none” for all the toxicological parameters (data not shown). Table 3 summarizes the compounds that show negligible or no undesired side effects, such as mutagenicity, tumorigenicity, irritant, and reproductive effects.

Among the CDs, we observed that chloramphenicol (DB214) shows high toxicity risk for all these toxicological properties. This observation builds on existing evidence that chloramphenicol is likely to have mutagenic, tumorigenic, irritant, and reproductive effects [43]. Toxicity has been estimated to be responsible for the attrition of approximately 33% of drug candidates, especially at the late stage of drug development. Therefore, early identification of potentially toxic chemotypes can help to circumvent safety liabilities [44].

### 2.3. Synthetic Accessibility Score

**Figure 12:**
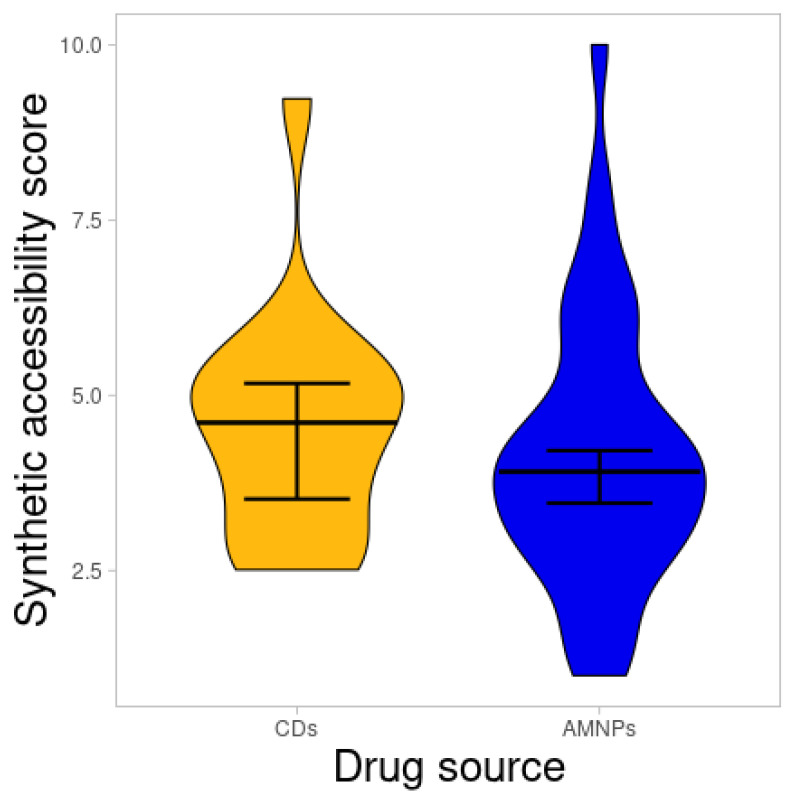
Violin plot of synthetic accessibility for CDs compared with AMNPs. The line that divides the boxes represents the median of the distributions. The black bars indicate a 95% confidence interval.

It is assumed that molecular fragments that frequently occur among easily obtainable compounds would be synthesized easily [45]. Hence, the synthetic accessibility score of AMNPs was predicted to know their ease of being produced. The result with a value of 1 (easy to synthesize) to 10 (not easy to synthesize) is presented below (Figure 12).

Similarly, there was no difference (*p* > 0.05) between the average synthetic accessibility score of the CDs and the AMNP categories (data not shown). However, most of the AMNPs accumulate closer to a synthetic accessibility score of 1 rather than 10, suggesting that most of the AMNPs could be synthesized with ease and that they are most unlikely to be rejected due to synthesis-related problems.

### 2.4. Hit Prioritization of AMNPs

**Figure 13:**
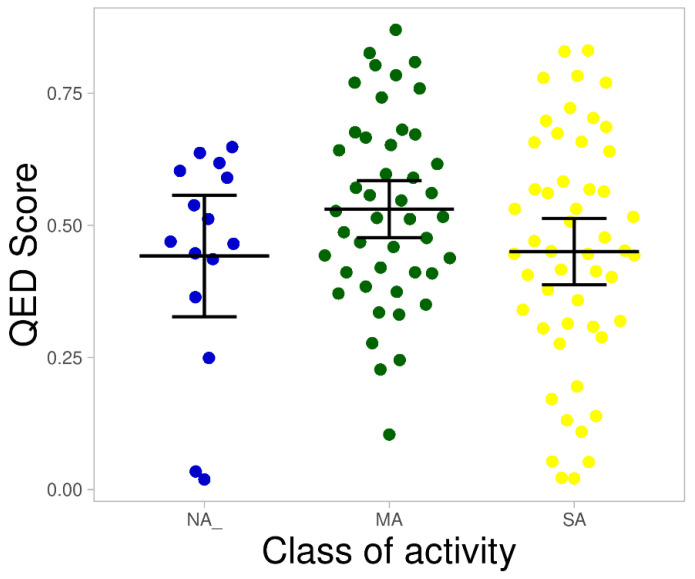
Distribution of the categories of AMNPs based on their desirability score. The markers with colors, red, green, and blue, represent MA, SA, and NA categories, respectively. The line that divides the boxes represents the median of the distributions. The black bars indicate a 95% confidence interval.

A balanced prioritization strategy that considers the physicochemistry and pharmacokinetics of bioactive compounds is vital for identifying molecules with the prospect to become candidate drugs [19,44,46]. In this study, quantitative estimation of drug-likeness (QED) described in Bickerton et al. [21] was used to prioritize the AMNPs. This was done by combining the desirability of key molecular descriptors (MW, TPSA, HBA, HBD, logP, RTBs, and aromatic rings, and ALERTs) into a single number that ranges between 0 and 1. A score of 1 in this context describes any compound with all its physicochemical descriptors within the space of an ideal drug-like profile. On the other hand, a score of 0 represents a compound with undesired properties. The visualization of the AMNPs based on these metrics is contained in Figure 13.

The result shows that most of the SA categories were distributed closer to 0 compared with others, which were distributed closer to 1. However, there was no significant difference between the average QED score of the various categories (MA = 0.53, NA = 0.44, and SA = 0.45). The compounds that showed a score closer to 1 among the SA subgroups may have more prospects for success during the preclinical drug discovery stage. On the other hand, compounds among the SA or MA categories that revealed a low QED score could be optimized to increase drug-likeness rather than potency. Similarly, those compounds that were found closer to 1 among the NA categories in Figure 13 might imply favorable drug-likeness, which may aid their success in the discovery pipelines. Thus, there might be a need to optimize more for potency among these compounds. A prioritized list of the AMNPs is shown in Figure S6, in the Supplementary Material. Wunberg et al. [46] conducted a data-driven screening of hits for drug-likeness and lead-likeness assessment. They established that the most promising molecules have good potency and liabilities that can easily be addressed. Therefore, some compounds with a poor balance of properties in the active categories might be a good starting point for discovery and design since most of them have room for optimization. Nevertheless, the identified liabilities of some of the AMNPs might vary in severity, thus complicating the optimization process.

### 2.5. Desirability Scoring Function Allows In Silico Hit Optimization Strategies

The goal of preclinical drug discovery is to maintain desirable properties with sufficient safety and improve on the identified liabilities in the lead compounds [47]. Therefore, molecules with deficiencies in their physicochemical descriptors or toxicological properties can be modified structurally for improvement [48]. In this study, two of the compounds from the SA categories that were identified with liabilities were optimized for a good balance of properties. The 2D structure of the compounds was drawn and modified using ChemDraw software (version 12.0), and their molecular descriptors were estimated using the MOE program (2019.01). The details of the selected compounds and processes are described below.

**Figure 14:**
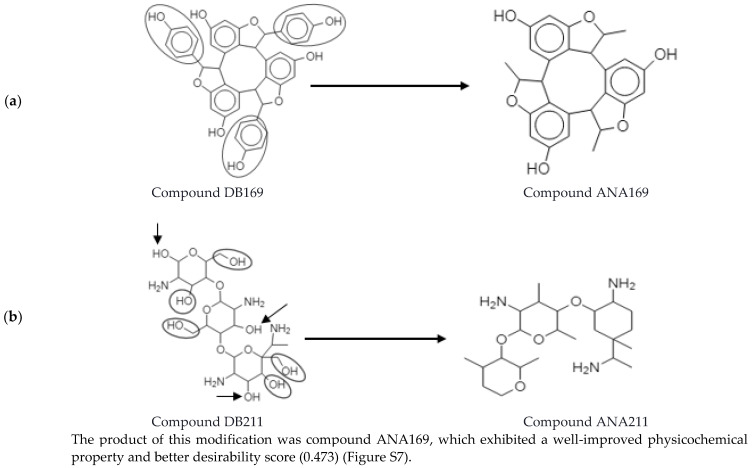
Optimization of some AMNPs with a low desirability score. (**a**) shows the random optimization of DB169 (α-viniferin) to produce ANA169. (**b**) shows the random optimization of DB211 (aminoethyl-chitosan) to produce ANA211. The round circles highlight the atoms that were replaced with a non-polar functional group (CH_3_), and the arrows are pointing to the atoms that were removed during optimization.

α-Viniferin (DB169) is one of the AMNPs that had a good potency (6.2 μM) but exhibited a low desirability score (0.143). Low absorption was one of the liabilities identified with this compound. It was thus optimized by replacing each of its phenolic components with a methyl (CH_3_) functional group (Figure 14a).

Similarly, aminoethyl-chitosan (DB211) is another compound that showed a good potency (0.147 μM) but a low desirability score (0.141). The poor pharmacokinetics and drug-likeness properties of this molecule were improved by removing or replacing some of its polar functional groups (OH and NH_2_). The details of this process are illustrated in Figure 14b. The functional groups highlighted in the compound DB211 represent the group of atoms that were strategically replaced (with a non-polar CH_3_ group) or removed during the optimization process.

The compound ANA211 obtained in this process has a well-improved property and better desirability score (0.808) (Figure S7, in the Supplementary Material). Interestingly, both ANA169 and ANA211 also showed better synthetic accessibility scores than their parent compounds (Figure S7). A search through the chemical database further revealed no result for both analogues, implying that they might be representing novel chemotypes.

The quest for minimizing the high attrition rates at the latter stages of drug development has necessitated the need to balance the efficacy of hit molecules with pharmacokinetic and toxicological properties through optimization [29,48,49]. In this study, the characterization of the drug-likeness profile of AMNPs has allowed us to propose and design multi-parameter hit optimization strategies for these compounds. This process is essential for building up activity against undesired effects and, at the same time, keeping the physicochemical properties in the drug-like range [50]. However, the bioactivity of the novel compounds obtained in this study was not assessed. Structure–activity relationship (SAR) or activity cliff will reveal which functional group is required for such bioactivity. Therefore, optimization should be done with a knowledge of the SAR.

### 2.6. Exploration of the Molecular Similarity/Diversity within the AMNPs

**Figure 15:**
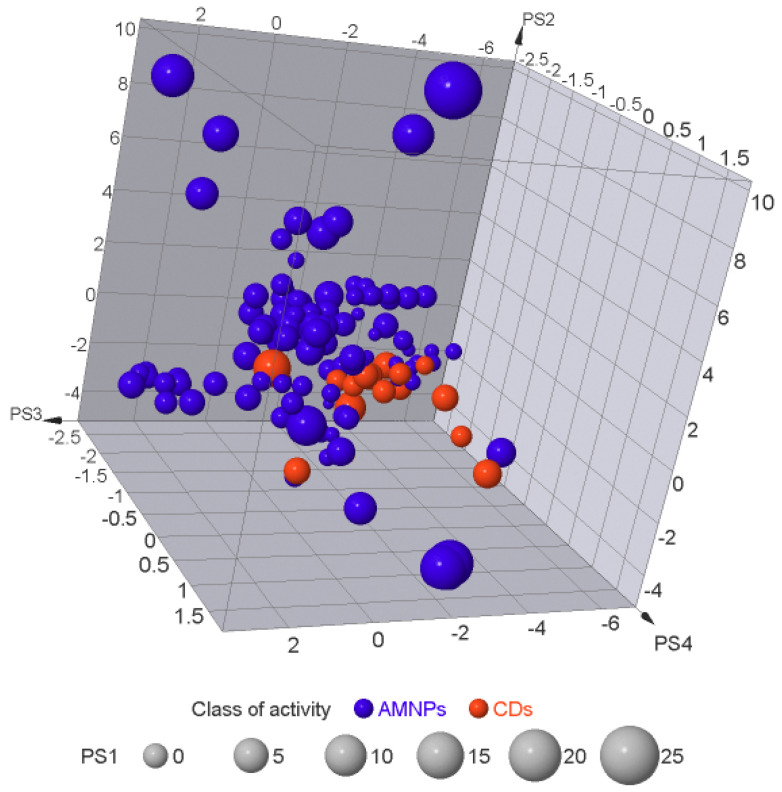
Distribution of AMNPs (blue markers) and CDs (red markers) in chemical space. The coordinates were generated from ChemGPS-NP with dimensions PS1, PS2, PS3, PS4 for size, aromaticity, lipophilicity, and flexibility, respectively. The AMNPs showed higher diversity in comparison with the CDs.

To visualize the chemical space occupied by the AMNPs relative to the CDs, principal component analysis (PCA) was conducted on the structural and physicochemical properties of the datasets. PCA is a statistical approach used to visualize molecular similarity/diversity within a molecule set [51]. It allows for the visualization of multidimensional data on two- or three-dimensional plots with reduced loss of information [51,52]. The result of the PCA for AMNPs relative to CDs is shown in Figure 15.

The first four components of the ChemGPS-NP Web map are interpreted as size, aromaticity, lipophilicity, and flexibility for PS1, PS2, PS3, and PS4, respectively. The *x*-, *y*-, and *z*-axes of the 3D plots are sPS2, PS3, and PS4, while PS1 represents the size of the markers. Although CDs were distributed across the chemical space, AMNPs showed a higher diversity (Figure 15). Furthermore, the plot showed that AMNPs were bigger than CDs (PS1), and a closer look at PS2 showed that AMNPs were more aromatic than CDs. Similarly, the plot on the *y*-axis reveals higher lipophilicity in favor of AMNPs, while the diversity of AMNPs tends towards less flexibility (PS4) compared with CDs. Regarding bioactivity, the significantly active (SA) category of the AMNPs was most diverse (data are not shown).

Based on the principle of medicinal chemistry that similar compounds bind to similar targets [52], few of the AMNPs that are highly far apart from the reference compounds (CDs) may indicate their prospect for novel mechanisms of actions. In light of the fact that most CDs are now known to be ineffective in treating MRSA infections, such active compounds with potentials for novel biological targets are highly desirable for anti-MRSA drug development.

Nevertheless, AMNPs situated closer to CDs in the chemical space may indicate similar pharmacokinetics or drug-likeness prospects. This might suggest that few of the AMNPs structurally related to CDs may have good pharmacokinetic properties and thus pass through preclinical screening for anti-MRSA drug development. The concepts of diversity and similarity of molecules are widely used in quantitative methods to design (selecting) a representative set of molecules and analyze the relationship between chemical structure and biological activity [16,51]. We further explored the relationship between the chemical structure and biological activity of the AMNPs in this study.

### 2.7. Structure–Activity Relationship (SAR) Landscape

**Figure 16:**
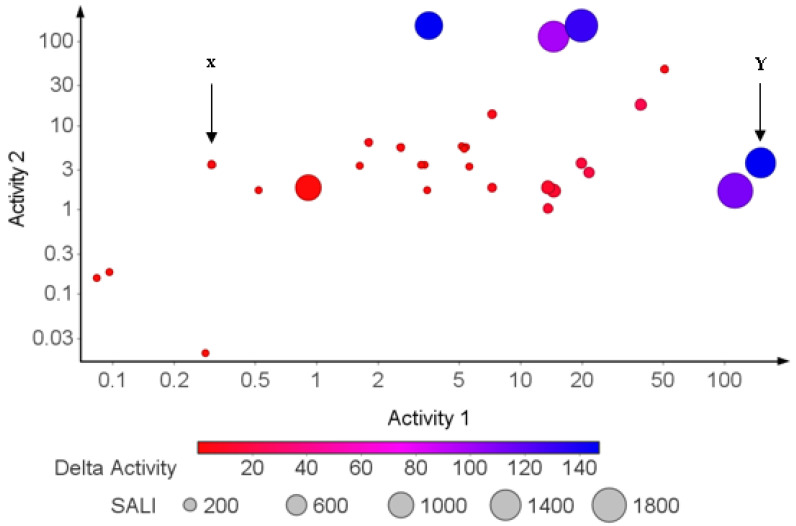
Scatter plot of the structure–activity relationship landscape of AMNPs. Markers were colored by delta activity and sized by SALI (structure–activity landscape index). Red markers (X) represent smooth regions of the SAR landscape. Pink, purple, and blue (Y) markers represent compounds that exhibited activity cliff.

The analysis of structure–activity relationships (SAR) is one of the fundamental tasks in medicinal chemistry. The underlying goal of exploring the SAR landscape is to identify structural differences between molecules that lead to differences in their bioactivities [53,54]. Therefore, given a pair of structurally similar molecules that showed activity cliff, the structure–activity landscape index (SALI) calculates how much potency is gained or lost, while delta activity represents the difference in the activity of a pair of similar molecules [16]. The results of the exploration of SAR for the AMNPs are summarized in Figure 16.

**Figure 17:**
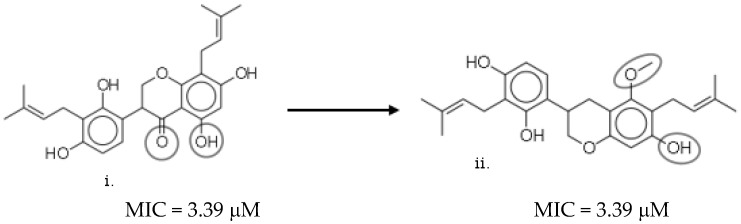
A pair of similar AMNPs (X) with little or no difference in bioactivity. The addition of hydroxyl (the small circle) in structure (ii) to the naphthalene ring in structure (i) did not cause any changes in bioactivity (MIC values). Hence, the two structures have about 87% structural similarity and the same bioactivity (delta activity = 0 µg/mL).

Red-colored markers represent pairs of similar compounds with little or no difference in bioactivity (see example in Figure 17). This is referred to as the smooth region (X) of the SAR landscape.

**Figure 18:**
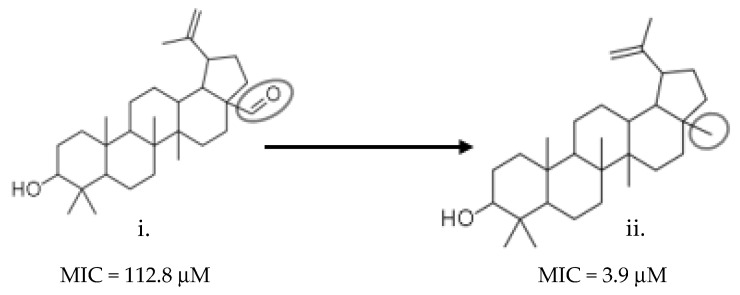
A pair of AMNPs (Y) showing an activity cliff. The two structures have about 94% similarity but are very widely different in bioactivity (delta activity = 256.1 µg/mL). An aldehyde group in structure (i) (the small circle) replaced by a methyl group in structure (ii) (the small circle) caused a significant difference in bioactivity.

Markers with pink, purple, and blue colors signify a pair of similar molecules with widely different bioactivities. These regions (e.g., Y) are called activity cliffs of the SAR landscape (Figure 18).

The concept of activity cliff is significant in identifying small structural modifications associated with large changes in potency [54]. Therefore, those AMNPs (markers identified with pink, purple, and blue colors, Figure 16) provide insight into potential functional groups in the AMNPs that can modulate bioactivities. This concept can be used by medicinal chemists who are interested in lead optimization projects. Conversely, the smooth region of the SAR landscape contains compounds that are good input data to build QSAR models [55] The predicted SAR between the AMNPs is provided in Table S2.

## 3. Materials and Methods

### 3.1. Data Collection and Preparation

An electronic search was conducted in May 2019 to identify relevant studies by employing freely available public databases (Google Scholar, ScienceDirect, Scopus, and PubMed). The keywords “marine OR natural products AND MRSA,” “phytochemicals AND MRSA,” and “MIC of phytochemicals AND MRSA” were used. The last search date was 20 May 2019. The reference lists of some of those eligible studies were also checked for related studies. Studies that reported the susceptibility of clinical isolates of MRSA to NPs, as determined by the reported minimum inhibitory concentration (MIC), were also included in this study. The search was customized and limited to reported publications from January 2009 to May 2019.

A total of 111 anti-MRSA natural products (AMNPs) with their in vitro bioactivity (Table S1) were retrieved based on the search strategy described above. The “simplified molecular input line entry system (SMILES)” structures of the AMNPs and their respective bioactivity data were stored as a text file. The reported MIC value of the different AMNPs (which ranged from 0.01 to 1600 μg/mL) was normalized. The datasets were divided into three categories using a modification of the previously described methods [56,57,58]. The normalized bioactivity categories include significantly active (SA) for MIC value below 2.5, moderately active (MA) for 2.5 < MIC ≤ 25, and negligibly active (NA) for MIC > 25. The 111 AMNPs were made up of 51 (45.9%) SA, 45 (40.5%) MA, and 15 (13.5%) NA. In addition, about 17 currently prescribed oral drugs for MRSA (CD) were also obtained from drug bank (https://go.drugbank.com/drugs) and used as the reference compounds (Figure S1).

### 3.2. Estimation of Molecular Descriptors and Physicochemical and Pharmacokinetic Properties of the Datasets

The SMILES structures of the datasets, retrieved from PubChem (http://pubchem.ncbi.nlm.nih.gov) [59], were uploaded onto the SwissADME [45] web server to estimate the physicochemical properties of AMNPs. Molecular descriptors such as molecular weight, hydrogen bonds, partition coefficient between n-octanol and water, rotatable bonds, and polar surface area were also predicted with the MOE program (2019.01) [60]. The mean values of these properties were calculated for the different bioactivity categories of AMNPs and compared with those of the current drugs for MRSA (CDs). The SwissADME web tool was used to predict the potential of each AMNP to inhibit the cytochrome P450 (CYP450) enzymes. Biotransformation processes of the compounds were predicted by using a freely available web service at www.biotransformer.ca (accessed on 22 November 2020) [61]. The rules proposed by Lipinski, Veber, and Egan were used to evaluate the drug-likeness of the AMNPs and CDs [45]. The absorption and bioavailability properties of AMNPs were also estimated as described by Daina and Zoete [35]. Toxicity properties such as mutagenic, tumorigenic, reproductive, and irritant effects were studied using Osiris DataWarrior software [62].

### 3.3. Exploration of Chemical Space (Chemical Diversity)

ChemGPS-NP Web was used to explore the chemical space occupied by AMNPs relative to CDs. The SMILES structure and identifier of the datasets were submitted in the space provided on the ChemGPS-NP Web service (http://chemgps.bmc.uu.se) [52]. The output with eight principal components added for each structure was retrieved as a text file. The text file was visualized on a 3D scatter plot in Osiris DataWarrior software [62] using the second (PS2), third (PS3), and fourth (PS4) principal components. Markers were colored according to the categories of bioactivity, and the first principal component (PS1) was used to size the markers.

### 3.4. Data Analysis and Visualization

Scatter plots, violin plots, and bar charts of the molecular descriptors, physicochemical properties, and other parameters estimated or predicted were plotted for AMNPs (and the SA, MA, and NA categories) and CDs using DataWarrior [62], PlotsOfData web tool [63], and Prism GraphPad 9.0 (GraphPad software). The mean of the molecular descriptors and physicochemical properties for AMNPs (and the SA, MA, and NA categories) and CDs were compared, and statistical differences were assessed with analysis of variance (ANOVA), with significance set at *p* < 0.05. Furthermore, the association between the in vitro activities (MIC) of AMNPs and the molecular descriptors or physicochemical properties was assessed using the Bravais–Pearson correlation coefficient (r).

## 4. Conclusions

This study was aimed at profiling natural products with their known in vitro activity against MRSA for drug-likeness and identifying their prospect for “hit”-to-“lead” optimization. Profiling of molecular descriptors and physicochemical properties of the datasets revealed that most of the AMNPs were not statistically different from the reference compounds (CDs). In addition, among the AMNP categories, SA was the most comparable to the reference compounds. Regarding drug-likeness, we observed that about 85% of the AMNPs obeyed Lipinski and Veber’s rule. There are greater chances for most of the AMNPs to achieve permeability at Veber’s limits than most of the currently prescribed anti-MRSA drugs. Evaluation of their pharmacokinetic properties showed that more than 77% of the AMNPs might have the prospect for good oral absorption, and most of these compounds are unlikely to be p-gp substrates.

Furthermore, about 34% of the AMNPs showed the prospect to become CNS drugs, an added advantage over the known anti-MRSA drugs. The few AMNPs predicted as non-inhibitors of CYP450 isoforms might be prioritized during hit selection. Toxicity profiling also revealed that about 60% of these compounds have negligible or no adverse effect. In addition, among the categories of AMNPs, NA exhibited the most promising prospect for oral drugs, reinforcing the idea that bioactivity does not guarantee that a compound will be useful. This result is relevant in drug discovery because poor physicochemical properties, pharmacokinetics, and high toxicity effects have continued to account for the most prominent cause of failure in drug discovery pipelines. Therefore, early information on potential drug candidates is crucial for saving time and economic costs of development [59,60]. This is because the process can potentially minimize the risk of future attrition. However, the lower compliance observed among SA and MA might necessitate optimizing more for their physicochemical properties towards balancing potency with efficacy.

The QED allows for the prioritization and identification of AMNPs with the desired drug-likeness. Interestingly, two of the compounds with identified drug-likeness deficiencies were enhanced by random replacements of some parts of their functional groups to evolve two promising and novel chemotypes with better synthetic accessibility scores. Exploration of the structure–activity relationship also revealed activity cliffs (i.e., similar compounds with significantly diverse activities), enabling the identification of chemical groups responsible for enhanced anti-MRSA bioactivity. Overall, this study could be used as a starting point in the pressing need for novel anti-MRSA therapies. Meanwhile, the antimicrobial activity of the novel chemotypes identified in this study was not ascertained. Furthermore, further in silico, in vitro, or in vivo studies are required to evaluate the prospective drug activity and efficacy of the prioritized hits.

## Figures and Tables

**Table 1:** The proportion of compounds that adhered to Lipinski’s rule when ≤ 1 of the parameters was violated.

Class of Activity	MW Violated	cLogP Violated	HBDs Violated	HBAs Violated
NA (*n* = 15)	12	13	12	12
MA (*n* = 45)	27	41	28	27
SA (*n* = 51)	25	41	26	25
AMNPs (*n* = 111)	64	95	66	64
CD (*n* = 17)	13	13	15	13

**Table 2:** Prediction of phase 1 and 2 biotransformations of AMNPs.

1	No. of AMNPs that Formed Metabolites (*n* = 111)
Phase 1	66
Phase 2	79
Both phase 1 and 2	56
Without Metabolites	22

**Table 3:** Summary of the estimated AMNPs and CDs with negligible or no undesired effects.

Class of Activity	Mutagenicity	Tumorigenicity	Reproductive Effects	Irritant Effects
CD (*n* = 17)	13	14	12	16
MA (*n* = 45)	40	41	36	38
NA (*n* = 15)	9	13	11	13
SA (*n* = 51)	44	49	37	46

## Data Availability

Not applicable.

## Supplementary Materials

The following are available online. Table S1: The selected AMNPs and their reported anti-MRSA activity. Figure S1: The chemical structures of the antibiotics that were used in the study. Figure S2: A scatter plot showing a weak negative correlation between cLogP and MIC values for the AMNPs. Figure S3: A scatter plot showing a strong positive correlation between TPSA and MW for the CDs. Figure S4: The BOILED-Egg predictive model for absorption and bioavailability of CDs. Figure S5: Toxicity profiling of AMNPs and CDs. Figure S6: The prioritized list of the AMNPs. Table S2: The estimated structure–activity relationship between the AMNP compounds.

## Sample Availability

Samples of the compounds are available from the authors.
