# Supplementary material for: Cheminformatic Profiling and Hit Prioritization of Natural Products with Activities against Methicillin-Resistant Staphylococcus aureus (MRSA)
Source: Molecules. 2021 Jun 16;26(12):3674. doi: 10.3390/molecules26123674 (PMC8246317; doi:10.3390/molecules26123674)
Supplement: Supplementary file 1 [file molecules-26-03674-s001.zip › molecules-1164853-supplementary.pdf]

Supplemental Data

Table S1 shows the selected natural products with their reported anti-MRSA activity.

SN	Natural compounds	Structure of the compounds	Source	Reported MIC values (µg/mL)	Average MIC values reported (µg/mL)	Normalized MIC (µM)	No of tested strains	Reference
DB101	Protosappanin B	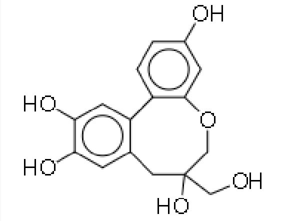	Plant (heart wood of *Caesalpinia sappan*)	128	128	38.95	10	(1)
DB102	Protosappanin A	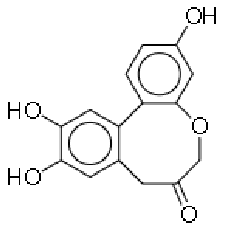	Plant (heart wood of *Caesalpinia sappan*)	64	64	17.42	10	(1)
DB103	Kuraridin	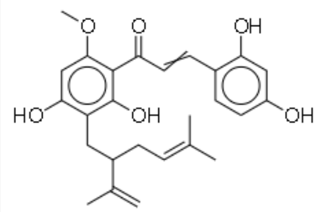	Root of *Sophora flavescens*	8	8	3.51	6	(2)
DB104	Oleanolic acids	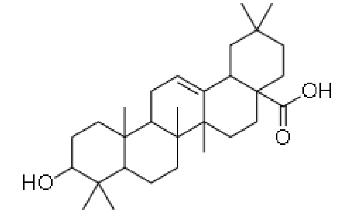	Plant (*Salvia officinalis)*	16-128	16	7.31	19	(3)

DB105	Licochalcone A	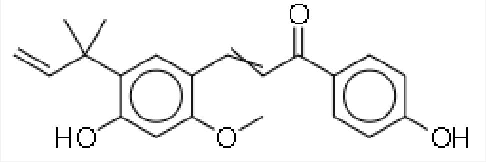	Plant (*Glycyrrhiza inflata*)	6.25	6.25	2.12	2	(4,5)
DB106	Chiricanine A	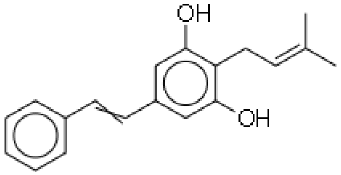	Extract of fungus-elicited peanuts (*Arachis hypogaea)*	12.5	12.5	3.50	1	(6)
DB107	Resveratrol	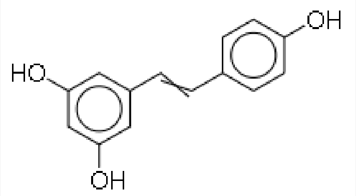	Fruits such as grapes peanuts and cranberries	1.25	1.25	0.29	3	(7)
DB108	Pterostilbene	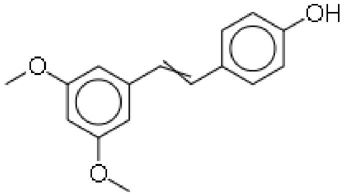	Fruits, majorly in Blueberries	0.078	0.078	0.02	2	(7)
DB109	Arahypin-5	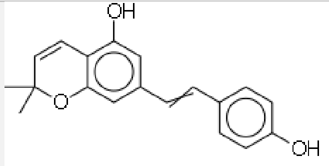	Plants (*Arachis hypogaea*)	25-50	25	7.36	1	(6)
DB110	Callislignan A	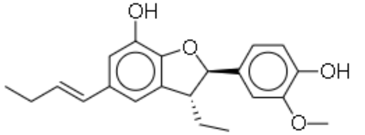	Shrub (*Callistemon lanceolatus*)	64	64	21.79	1	(8)
DB111	Callislignan B	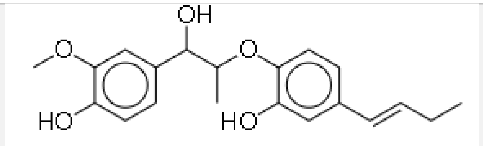	Shrub (*Callistemon lanceolatus)*	8	8	2.76	1	(8)

DB112	Thymoquinone	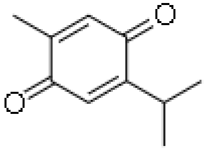	Medicinal herb; (*Nigella sativa*)	8-16	16	2.63	12	(9)
DB113	Myricetin	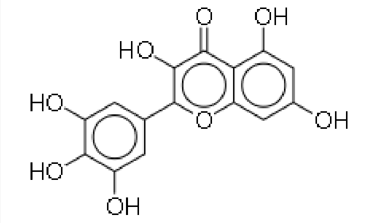	Plant (Guss) extract	62.5	62.5	19.89	1	(10)
DB114	Luteolin	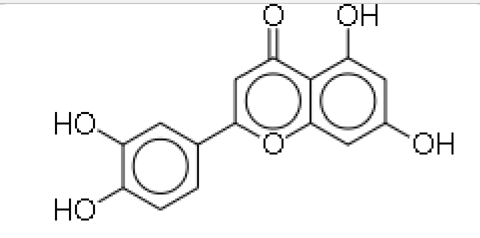	Plant (Guss) extract	31.2-125	125	3.58	29	(10,11)
DB115	Friedelin	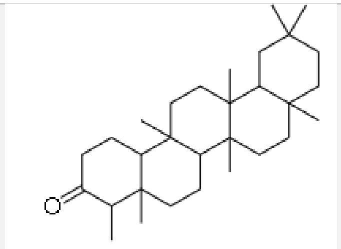	Plants (*Pterocarpus santalinoides*)	10	10	4.27	NA	(12)
DB116	7-hydroxycoumarin	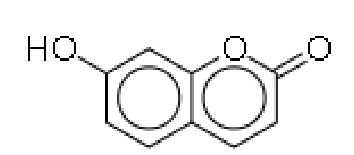	Sigma	200	200	32.42	1	(13)
DB117	Indole-3-Carbinol	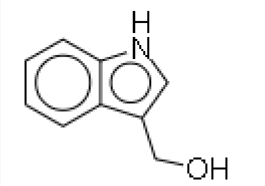	Sigma	400	400	58.87	1	(13)

DB118	Salicyclic acid	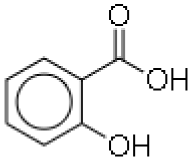	Sigma	1600	1600	220.99	1	(13)
DB119	Gallic acid	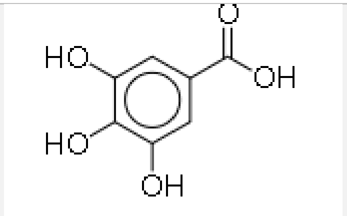	Honey and plants (such as *Bauhinia kockiana*)	600	600	102.07	1	(14)
DB120	Methyl gallate (methyl 3,4,5-trihydroxybenzoate)	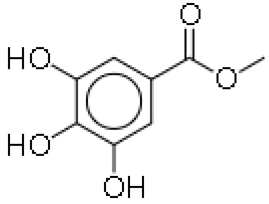	*Bauhinia kockiana*	500	250	46.04	1	(14)
DB121	Ferulic acid	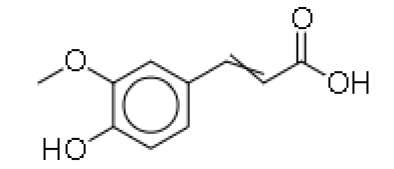	Mushrooms	0.5	0.5	0.10	1	(15)
DB122	Quercetin	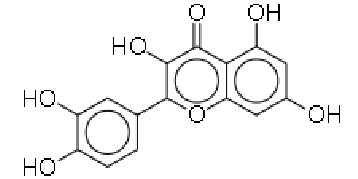	Plants (Guss extracts)	500	500	151.12	11	(16)
DB123	Watasemycin A	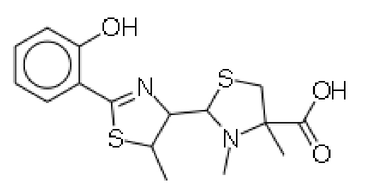	Streptomyces sp. OUCMDZ-1703	7.81	7.81	2.75	3	(17)

DB124	Dulcisxanthone J (Cudraxanthone B)	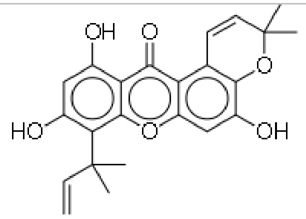	Medicinal plant (*Garcinia dulcis)*	16	16	6.31	1	(18)
DB125	12b-Hydroxy-des-D-garcigerrin A	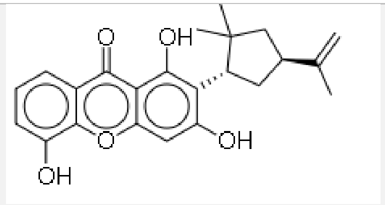	Medicinal plant (*Garcinia dulcis)*	4	4	1.52	1	(18)
DB126	(6*E*,12*E*)-tetradecadiene-8,10-diyne-1,3-diol	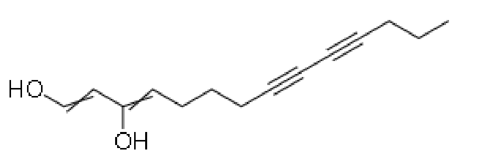	Plant (*Atractylodes japonica*)	4-32	8	1.75	12	(19)
DB127	Panduratin A	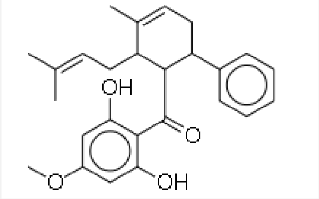	Rhizome of fingerroot (*Kaempferia pandurate)*	1	1	0.41	27	(20)
DB128	Atractylenolide III	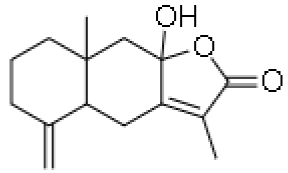	Plants (*Atractylodes japonica*)	8-128	32	7.95	12	(19)
DB129	Atractylenolide I	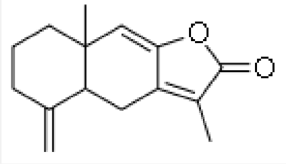	Plant (*Atractylodes japonica*)	8 -128	64	14.74	12	(19)

DB130	Carnosic acid	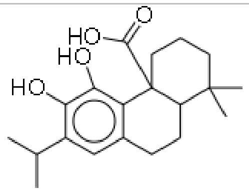	From leaves of plant (*Rosmarinus officinalis)*	12-16	16	5.32	3	(21)
DB131	Juncusol	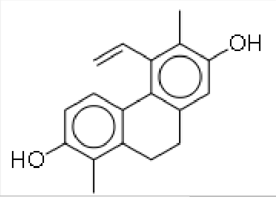	Plants (Juncaceae family)	25	16	4.26	1	(22)
DB132	Juncuenin D	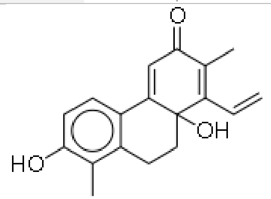	Plants (Juncaceae family)	12.5	12.5	3.53	1	(22)
DB133	Caesalfurfuric acid A	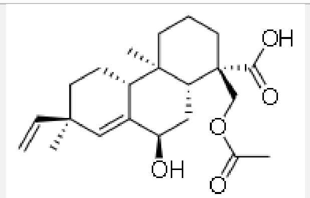	Twig of plant (*Caesalpinia furfuracea*)	16	16	6.02	1	(23)
DB134	Dihydrokaempferol	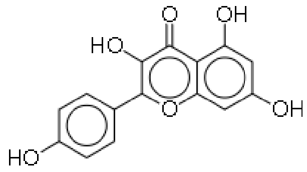	Plant (*Commiphora pedunculata*)	12.5	12.5	3.58	1	(24)
DB135	Sanguinarine	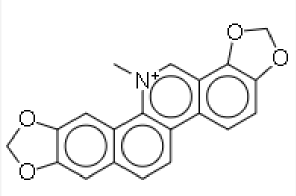	Plants (Papaveraceae families)	3.12- 6.25	3.12	1.04	15	(25)

DB136	α- Amyrin	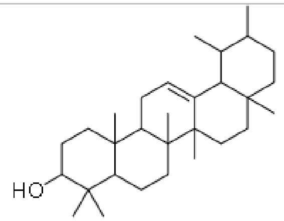	Plants (bark of *Callicarpa farinosa*)	2-64	32	13.66	17	(26)
DB137	Betulinic acid	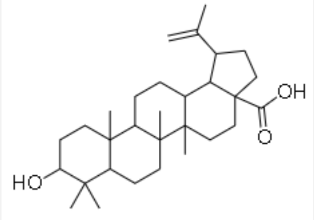	Plant (bark of *Callicarpa farinosa*)	8-64	32	14.61	17	(26)
DB138	Betulinaldehyde	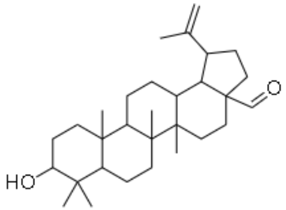	Plant (bark of *Callicarpa farinosa*)	8-512	256	112.82	17	(26)
DB139	Carnosol	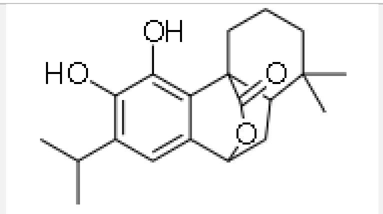	*Plants (Rosmarinus officinalis)*	16	16	5.29	3	(27)
DB140	8-hydroxy-9, 10-diisobutyloxythymol	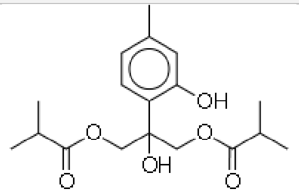	Roots of *Inula hupehensis*	62.8	62.8	21.25	NA	(28)
DB141	Abyssomicin C	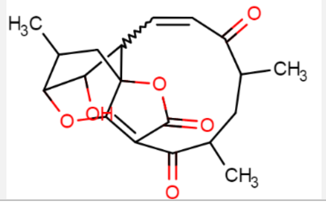	Marine bacteria (Verrucosispora AB-18-032)	4	4	1.385	1	(29)

DB142	MC21-A(Bromophene)	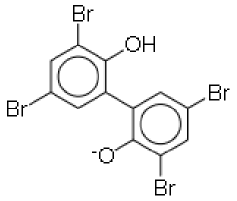	Marine bacterium (*Pseudoalteromonas phenolica*)	1–2	1	0.50	10	(29)
DB143	α-Pyrone-I	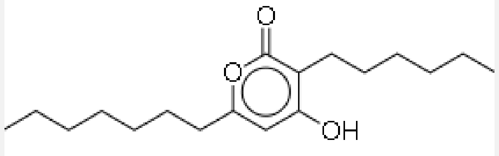	Marine bacterium (Pseudomonas sp. F92S91)	4	4	1.18	NA	(29)
DB144	Erybraedin A	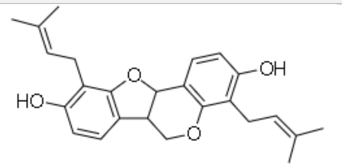	Plants (Erythrina spp)	0.78-3.13	0.78	0.31	4	(30)
DB145	Thiomarinol A	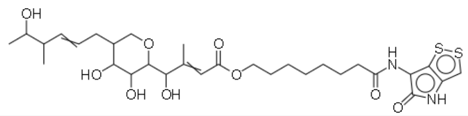	Marine bacterium (*Alteromonas rava* SANK73390)	≤0.01	0.01	0.001	1	(29)
DB146	Andrimid	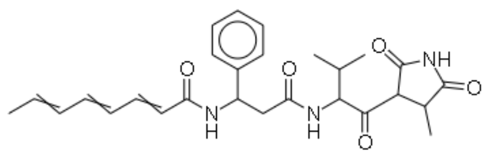	Marine bacterium (*Pseudomonas fluorescens*)	2	2	0.96		(29)
DB147	Etamycin A (Viridogrisein)	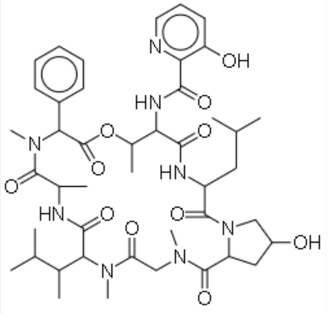	Marine bacterium (Streptomyces sp. CNS-575)	4-16	16	14.06	3	(31)

DB148	Pestalone	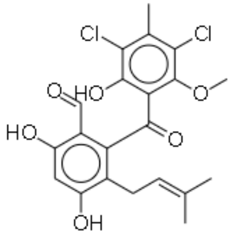	Marine fungus (*Pestalotia sp.*)	0.037	0.037	0.02	NA	(29)
DB149	Apigenin	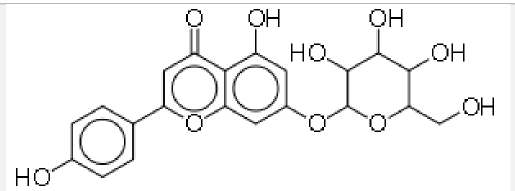	Plants (*Scutellaria barbata*)	3.9–15.6	3.9	1.69	20	(30)
DB150	Heyneanol A	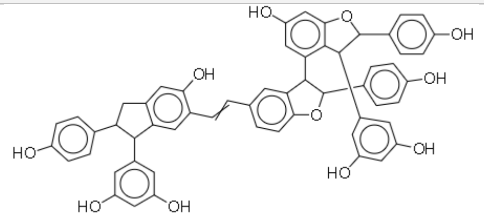	Grape vine (*Vitis spp.*)	2	2	1,8099	4	(30)
DB151	Erycristagallin	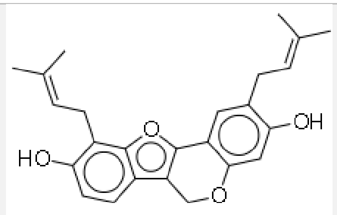	Stem of plants (*Erythrina subumbrans*)	0.78-1.56	0.78	0.30	4	(30)
DB152	Acylphloroglucinol	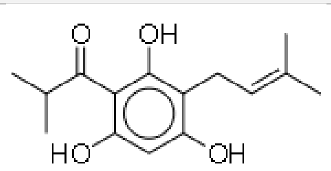	Plants (*Helichrysum gymnocomum*)	7.8	7.8	2.06	1	(30)

DB153	Hardwickiic acid	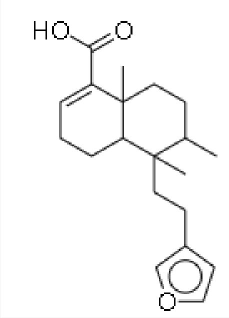	Stem bark of plants (*Irvingia gabonensis*)	19.53	19.53	6.17	1	(30)
DB154	Mangostanin	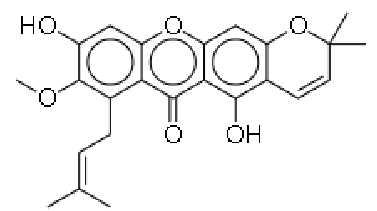	Fruit (*Garcinia cowa*)	4.0	4.0	1.63	1	(30)
DB155	α-mangostin	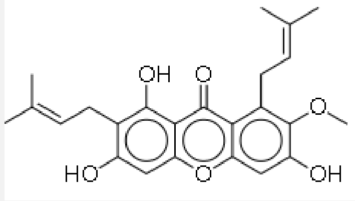	Fruit (*Garcinia cowa*)	8.0	8.0	3.28	1	(30)
DB156	Tripropeptin D	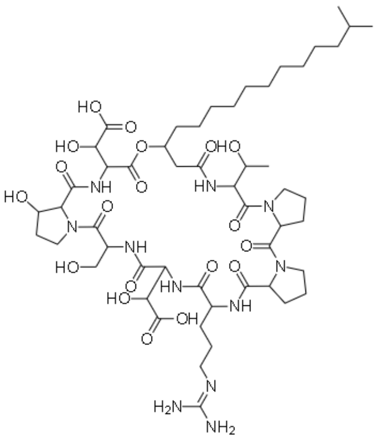	Culture of bacteria (*Lysobacter sp*).	0.78–1.56	0.78	0.91	8	(30)

DB157	Zeylasterone	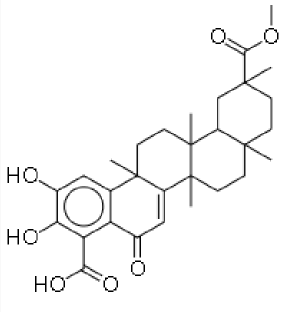	Plants (*Maytenus blepharodes*)	5-10	10.5	5.36	1	(32,33)
DB158	Tripropeptin C	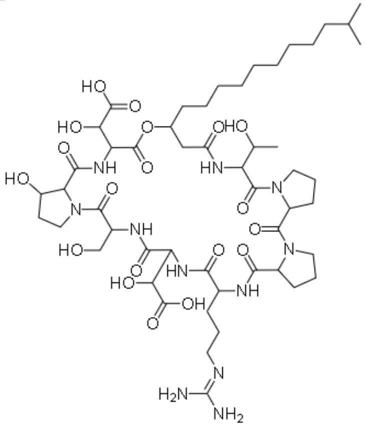	Culture of *Lysobacter sp.*	1.56–3.12	1.56	1.80	8	(30)
DB159	Myrifabine	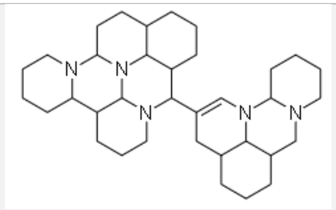	*Myrioneuron faberi*	6.32	6.32	3.45	1	(34)
DB160	Baicalein	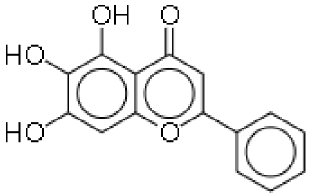	Herbal plants	128	128	34.59	6	(2)

DB161	Asphodoside B	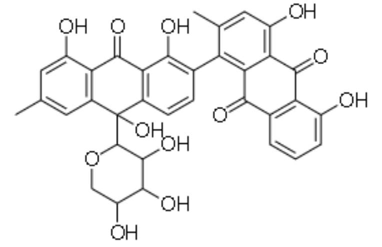	Plants (*Asphodelus microcarpus*)	1.62	1.62	1.04	1	(35)
DB162	Bartericin A	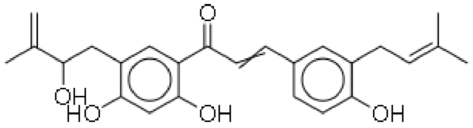	Plant (*Dorstenia angusticornis*)	0.61	0.61	0.25	1	(4)
DB163	Anthocyanin	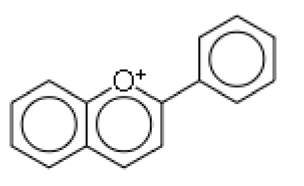	Plant (*Clerodendron infortunatum*)	1.6	1.6	0.33	1	(36)
DB164	Corylifol C	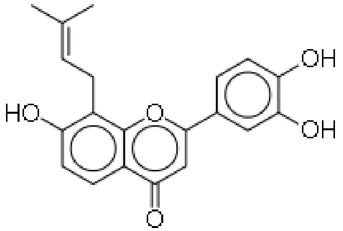	Plant (*Psoralea corylifolia*)	16	16	5.41	3	(37)
DB165	Neobavaisoflavone	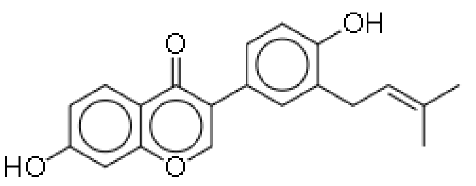	Plant (*Psoralea corylifolia* fruits)	16	16	5.16	2	(37)
DB166	Isobavachalcone	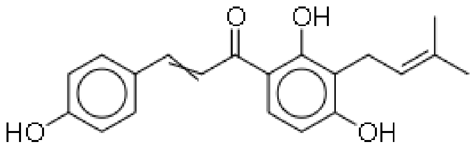	Plant (*Psoralea corylifolia* fruits)	8	8	2.59	2	(37)

DB167	Corylifol B	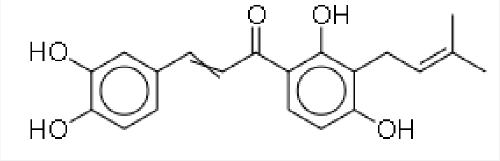	Plant (*Psoralea corylifolia* fruits)	16	16	5.45	2	(37)
DB168	Bakuchiol	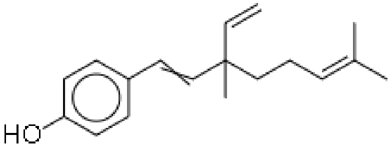	Plant (*Psoralea corylifoli*)	8	8	2.05	2	(37)
DB169	Alpha -viniferin	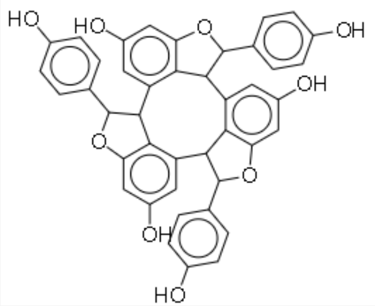	Medicinal plants (*Carex humilis*)	6.24-12.49	6.24	6.24	2	(38)
DB170	Tannic acid	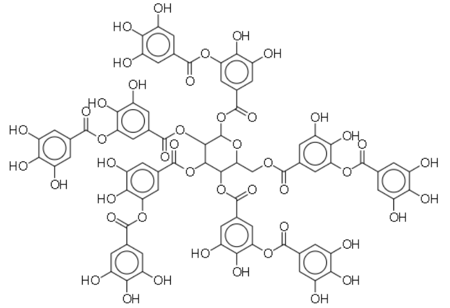	Plant (*Bauhinia kockiana*)	256	256	435.51	3	(14,39)
DB171	Sorocenol G	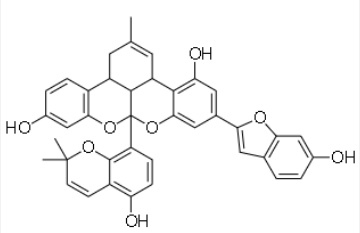	*Sorocea muriculata*	0.94	0.94	0.59	1	(35)

DB172	Carvacrol	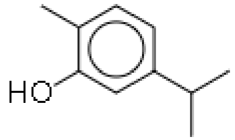	Plants	300	300	45.07	3	(40)
DB173	Benzyl isothiocyanate (BITC)	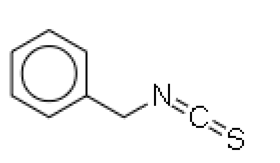	Plants	200	200	29,842	3	(40)
DB174	Berberine	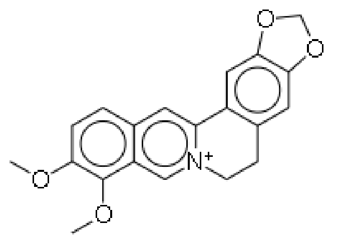	Plants	40	40	13.45	1	(40)
DB175	Punicalagin	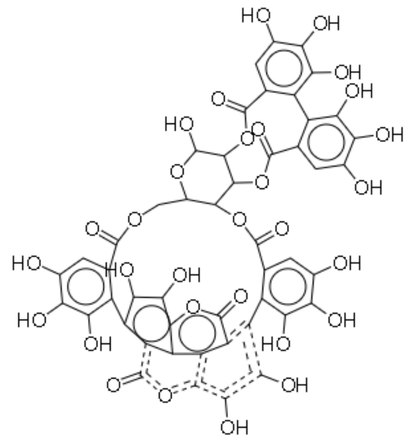	Pomegranate fruits	61.5	61.5	66.71	NA	(41)
DB176	Vanillic acid	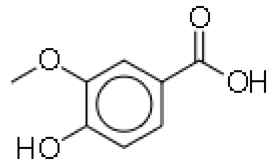	Mushroom	0.5	0.5	0.08	NA	(15)

DB177	2,4-dihydroxybenzoic	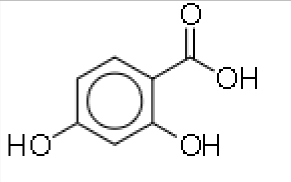	Mushroom	0.5	0.5	0.08	NA	(15)
DB178	ϱ-coumaric (4-Hydroxycinnamic acid)	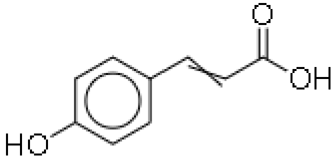	Mushroom	1	1	0.16	NA	(15)
DB179	Syringic	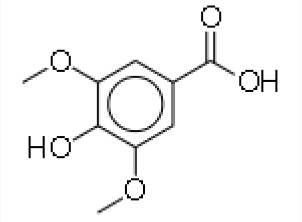	Mushroom	0.5	0.5	0.01	NA	(15)
DB180	Protocatechuic acid	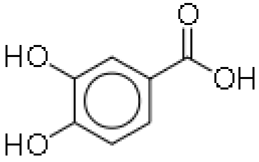	Plants and mushroom	1	1	0.15	1	(15)
DB181	Decyl gallate	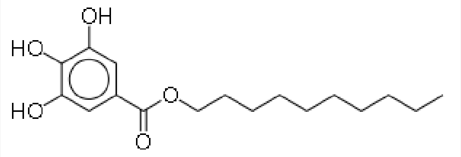	Plants	12.5	12.5	3.88	1	(14)
DB182	Ursolic acid	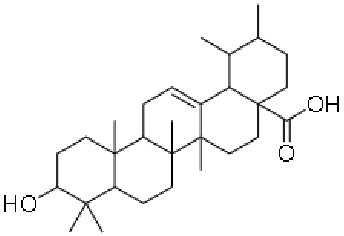	Plant (*Salvia officinalis*)	4-8	4	1.83	19	(3,30)

DB183	Demethyltexasin	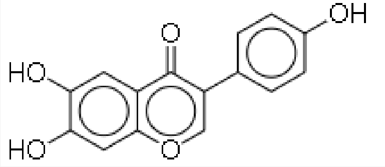	Soya beans	16-64	64	17.30	7	(42)
DB184	4-(((Z)-5-((4-((E)-3-(2-chlorophenyl)-3-oxoprop-1-en-1-yl) benzylidene)-2,4 dioxothiazolidin-3-yl)methyl)benzoic acid	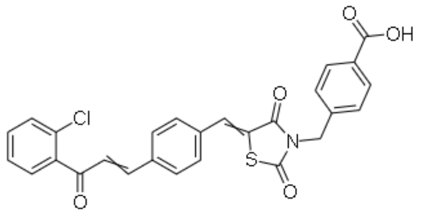	Chalcone derivative	0.5-1	1	0.50	2	(43)
DB185	Gancaonin G	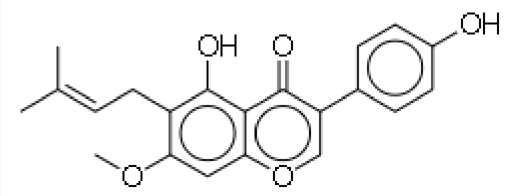	Plant (*Glycyrrhiza uralensis*)	16	16	5.64	4	(37)
DB186	3′-(γ,γ-dimethylallyl)-kievitone	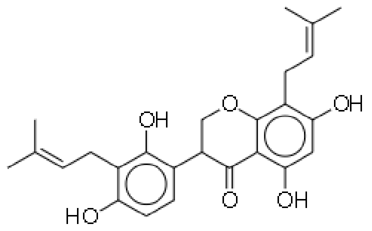	Plant (*Glycyrrhuralensis)*	8	8	3.40	4	(37)
DB187	8-(γ,γ-dimethylallyl)-wighteone,	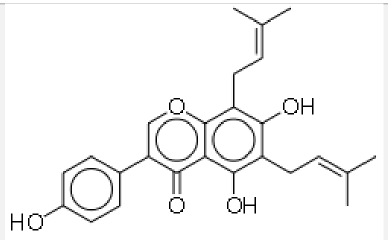	Plant (*Glycyrrhiza uralensis)*	8	8	3.25	4	(37)

DB188	Myoporumine A	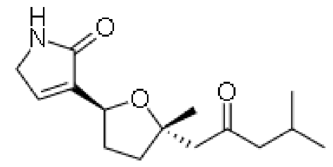	Semi mangrove plant (*Myoporum bontioides)*	6.25	6.25	1.66	1	(44)
DB189	Glabridin	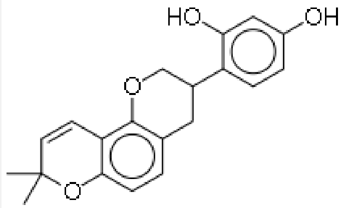	Plant (*Glycyrrhiza uralensis*)	16	16	5.18	4	(37)
DB190	Lupeol	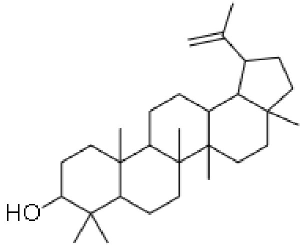	Plants (*Acokanthera oppositifolia*)	3.9	3.9	1.66	1	(45)
DB191	epsilon-Viniferin	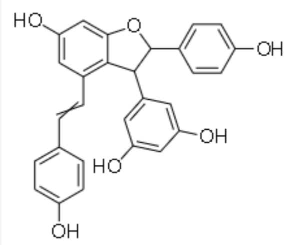	Plant (*Shorea maxwelliana)*	100	100	45.45	2	(46)
DB192	5-geranyloxy-7-methoxycoumarin	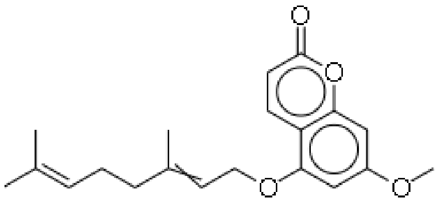	Plants (*Zanthoxylum nitidum*)	8-32	16	5.25	4	(47)

DB193	Mammea A/BA	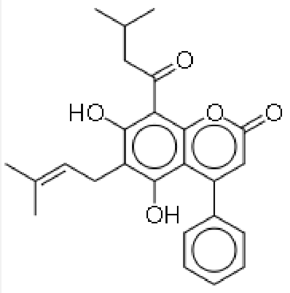	22 different Mexican medicinal plants	2	2	0.813	2	(47)
DB194	Rugulosin A	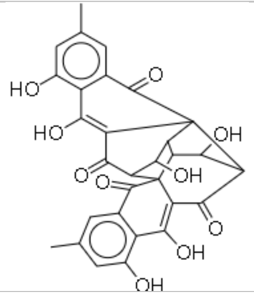	*Penicillium radicum FKI-3765-2*	0.125	0.125	0.07	1	(48)
DB195	Sepicanin A (2(S)-6-Geranyl-5,7,20,40-tetrahydroxy-flavanone)	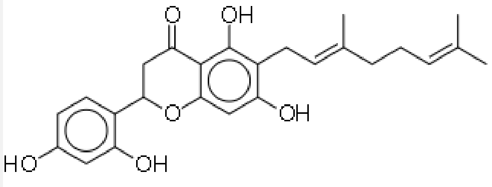	Plant (*Artocarpus sepicanus*)	1.23	1.2	0.52	1	(49)
DB196	Cryptotanshinon	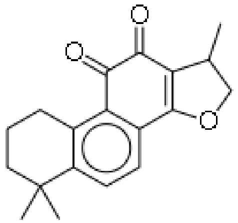	Root of plants (*Salvia miltiorrhiza*)	0.5-8	2	0.59	16	(50)
DB197	Stigmasterol	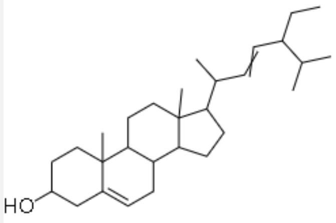	Stem bark of *Neocarya macrophylla*	6.25	6.25	2.58	1	(51)

DB198	Myoporumine B	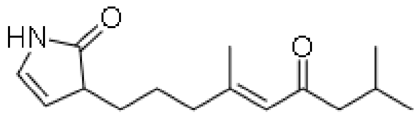	Semi mangrove plant (*Myoporum bontioides)*	6.25	6.25	1.56	1	(44)
DB199	Hinokinin	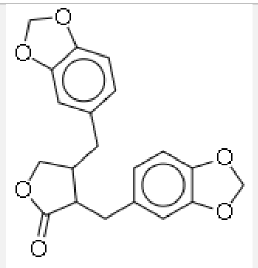	Plant (*Commiphora leptophloeos*)	0.0485-3.125	1.560	0.55	4	(52)
DB200	Curcumin	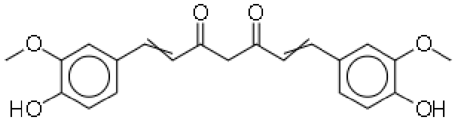	Inhibition of growth via perturbation of bacterial membrane integrity	125-250	250	92.10	4	(53)
DB201	Talaromannin A	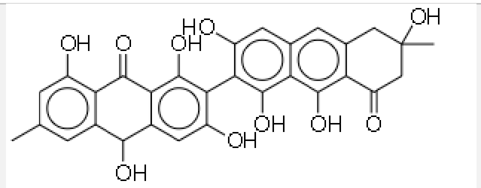	Fungus (*Talaromyces wortmannii*)	4	4	2.18	1	(54)
DB202	Emodin	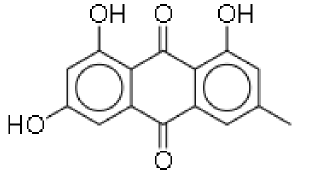	Fungus (*Talaromyces wortmannii*)	4	4	1.08	1	(54)
DB203	Caffeic acid	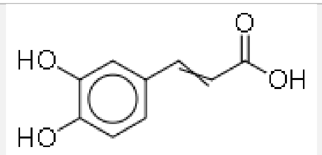	Wild mushroom	1	1	0.18	1	(55)

DB204	Sophoraflavanone G	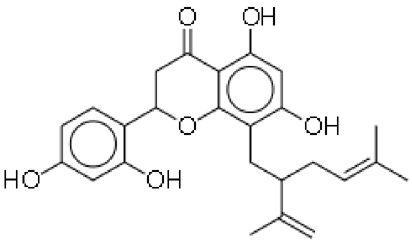	Root of *Sophora flavescens*	4	4	1.70	6	(2)
DB205	Artanin	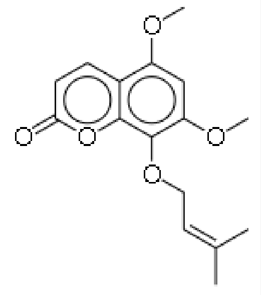	Plants (*Zanthoxylum nitidum*)	8-64	16	4.64	4	(47)
DB206	Aerugine	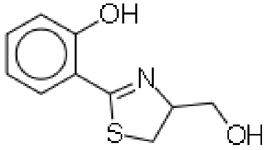	Streptomyces sp. OUCMDZ-1703	7.81	7.81	1.63	3	(17)
DB207	Ikarugamycin	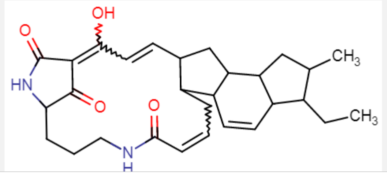	*Streptomyces zhaozhouensis* CA-185989	2-4	2	0.96	1	(56)
DB208	Licoricidin	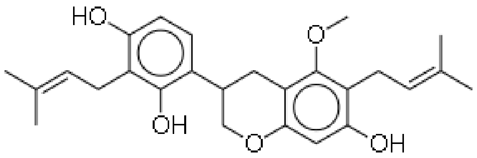	Fruits (*Psoralea corylifolia)*	8	8	3.40	3	(37)
DB209	Acetyl-11-keto-b-boswellic acid	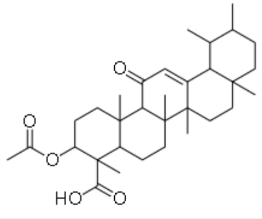	Plant (*Boswellia serrata*)	2-4	2	1.03	50	(57)

DB210	Celastrol	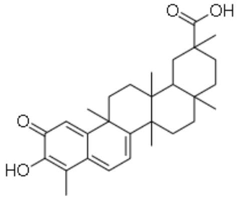	The root extract of plants (*Tripterygium wilfordii*)	2	2	0.90	1	(58)
DB211	Aminoethyl-chitosan	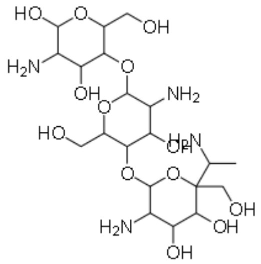	Chitin	0.5	0.5	0.15	6	(59)

Figure S1. Chemical structures of the antibiotics that were used in the study.

DB212 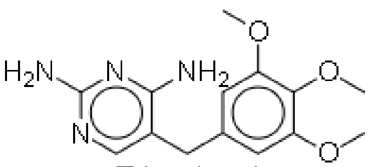 Trimethoprim	DB213 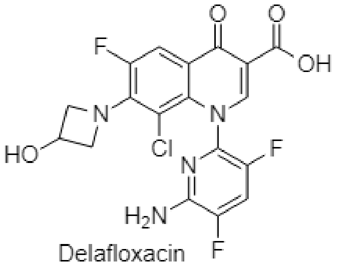 Delafloxacin	DB 214 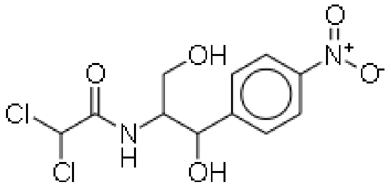 Chloramphenicol
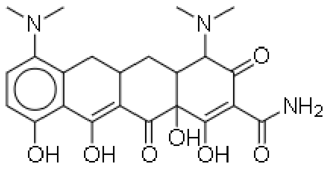 Minocycline DB215	DB216 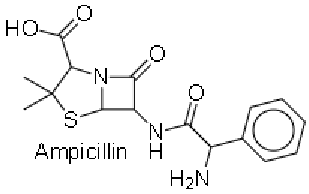 Ampicillin	DB217 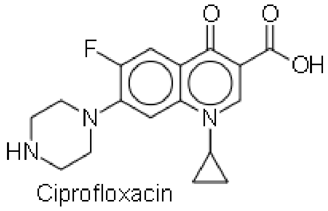 Ciprofloxacin
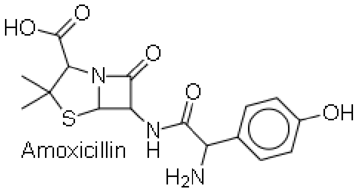 Amoxicillin DB218	DB219 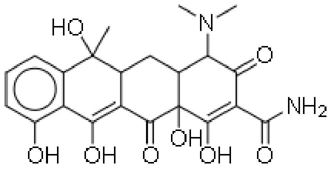 Tetracycline	DB220 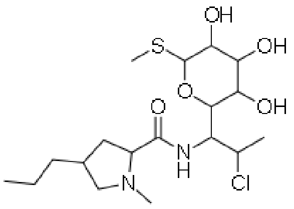 Clindamycin
DB221 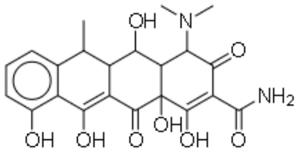 Doxycycline	DB222 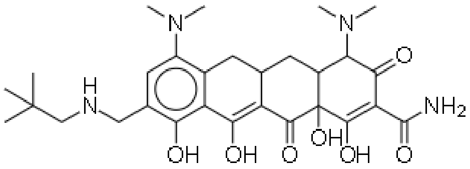 Omadacycline	DB223 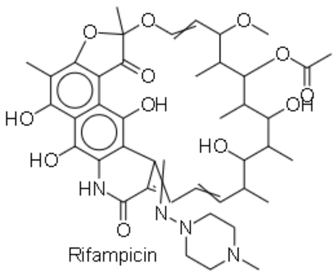 Rifampicin
DB224 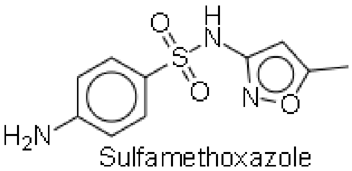 Sulfamethoxazole	DB225 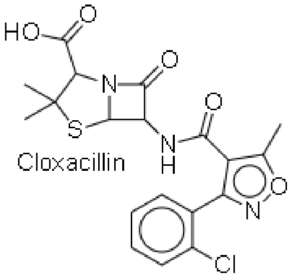 Cloxacillin	DB226 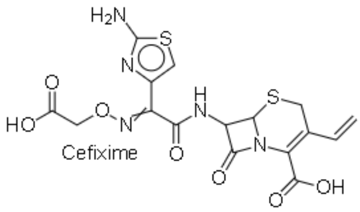 Cefixime
DB227 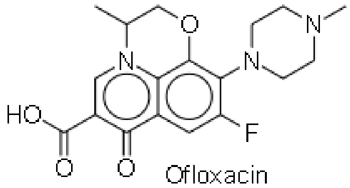 Ofloxacin	DB228 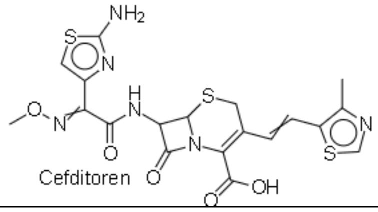 Cefditoren	

Figure S2. Scatter plot showing a weak negative correlation between cLogP and MIC values for the AMNPs.

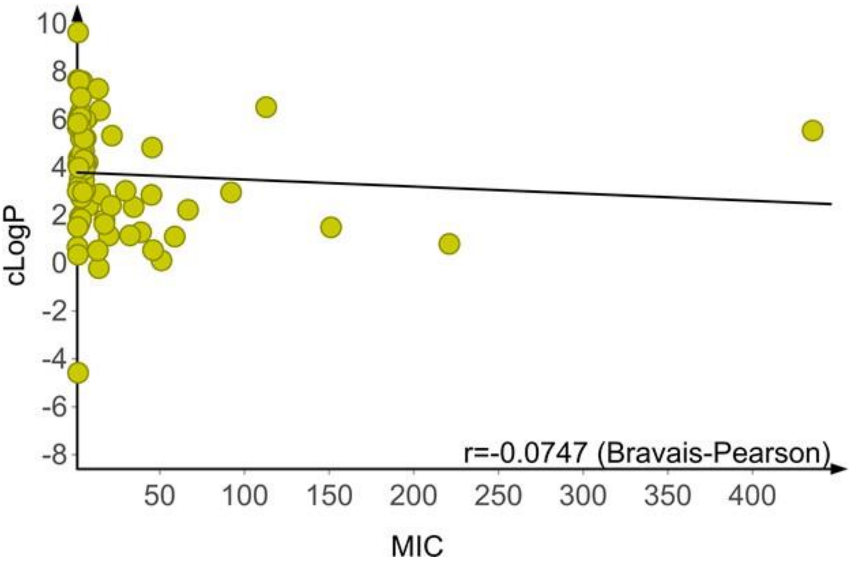

Figure S3. Scatter plot showing a strong positive correlation between TPSA and MW for the CDs

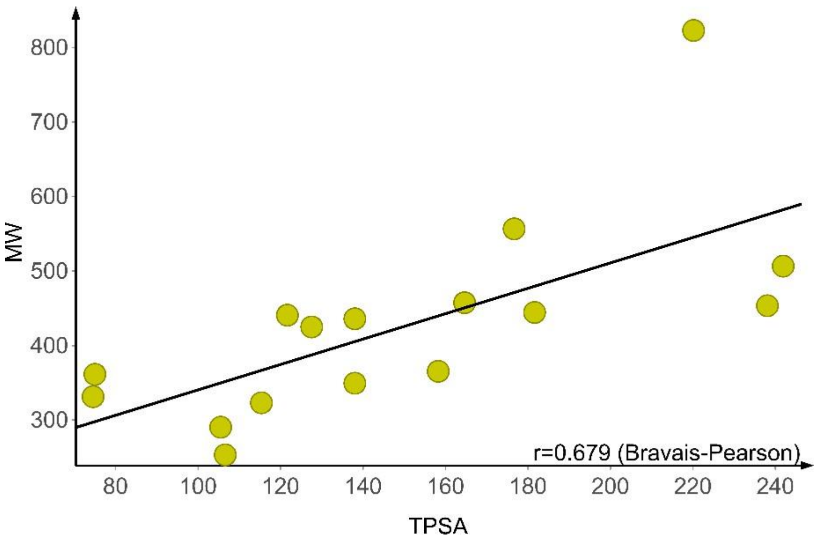

Figure S4. BOILED-Egg predictive model for absorption and bioavailability of CDs. The markers (representing CDs) within the white region are occupied by molecules (45 %) that are most likely absorbed by the gastrointestinal tract. The yellow region is the area occupied by potential CNS drugs. The blue and red markers respectively represent substrates and non-substrates of p-gp. The few markers outside the white region, and three others that are out of the range of this model (not shown), represent the poorly absorbed drug compounds.

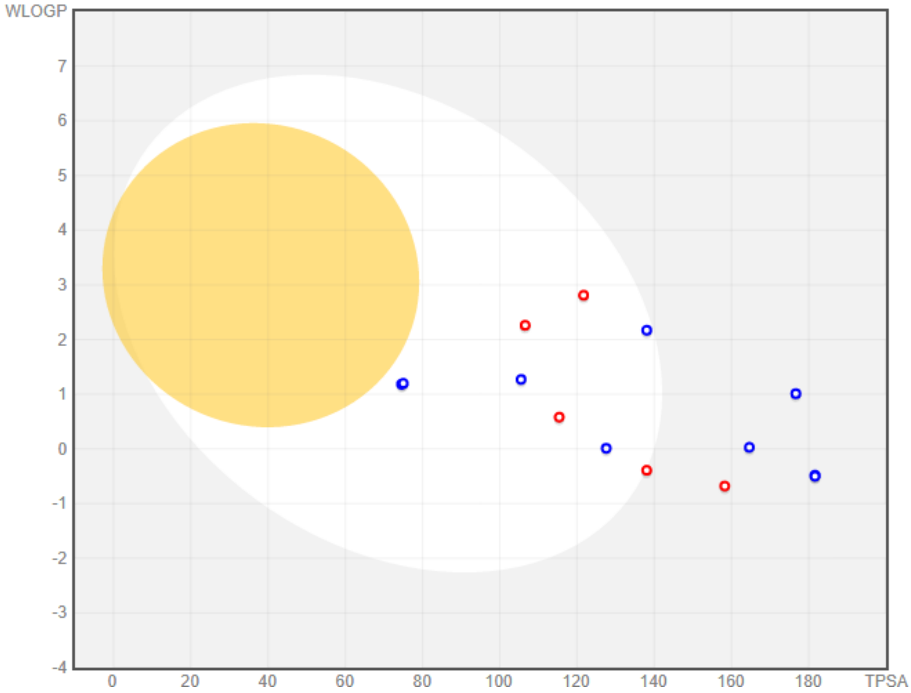

Figure S5. Toxicity profiling of AMNPs and CDs. About 59 % and 80 % of AMNPs and CDs, respectively, may likelyhave negligible or no toxicological effects.

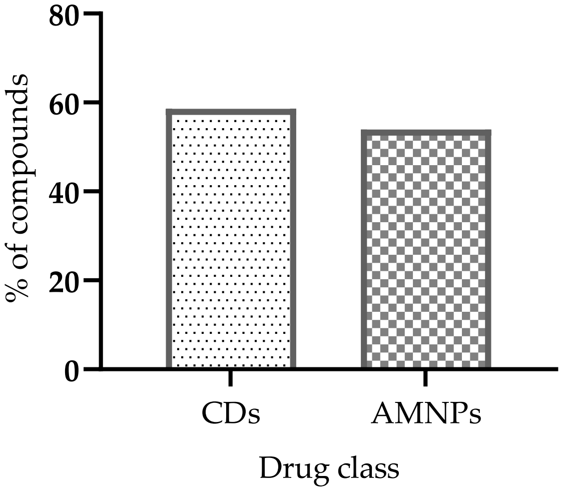

Figure S6. Prioritized list of the AMNPs

ID	Structure of Name of compounds	Class of activity	MIC(□M)	QED
DB123	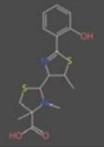	MA	2.7527907	0.870
DB188	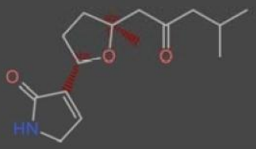	SA	1.6584375	0.831
DB108	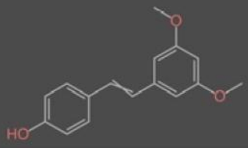	SA	0.0199914	0.829
DB132	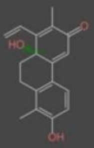	MA	3.529125	0.826
DB153	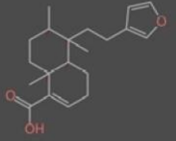	MA	6.1798779	0.809
DB189	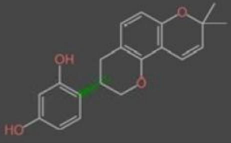	MA	5.18992	0.803
DB131	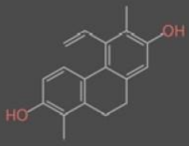	MA	4.26128	0.784
DB199	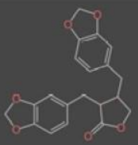	SA	0.552786	0.783
DB206	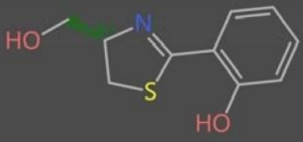	SA	1.6343206	0.779
DB179	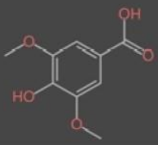	SA	0.099085	0.770
DB109	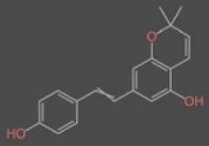	MA	7.3585	0.770
DB110	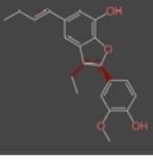	MA	21.78624	0.759

ID	Structure of Name of compounds	Class of activity	MIC(□M)	QED
DB140	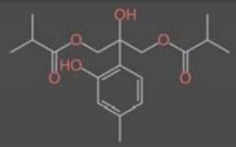	MA	21.25152	0.742
DB121	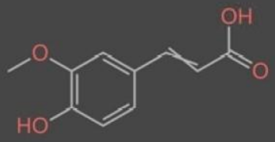	SA	0.09709	0.722
DB176	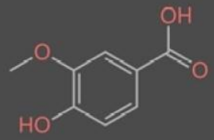	SA	0.084075	0.703
DB198	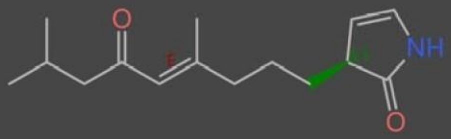	SA	1.5584375	0.698
DB107	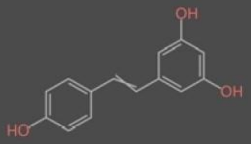	SA	0.2853	0.686
DB111	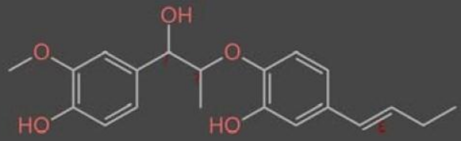	MA	2.7552	0.681
DB130	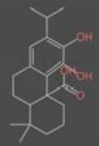	MA	5.31888	0.676
DB196	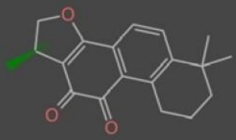	SA	0.59272	0.674
DB165	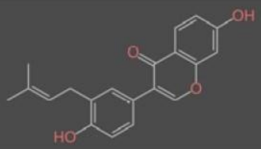	MA	5.1576	0.672
DB174	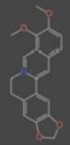	MA	13.4544	0.666
DB168	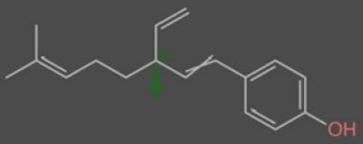	SA	2.05104	0.658
DB178	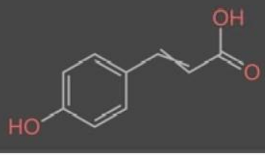	SA	0.16416	0.657

ID	Structure of Name of compounds	Class of activity	MIC(□M)	QED
DB185	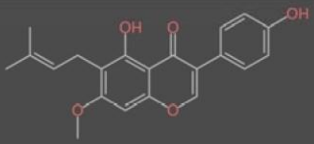	MA	5.63808	0.652
DB172	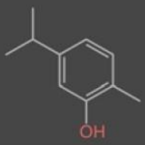	NA	45.066	0.648
DB102	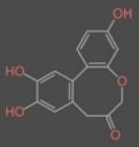	MA	17.424	0.642
DB144	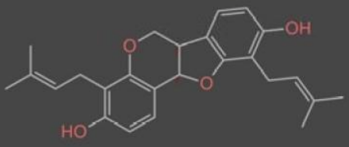	SA	0.3061422	0.640
DB117	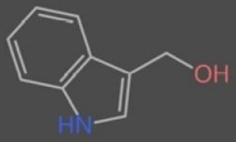	NA	58.868	0.637
DB118	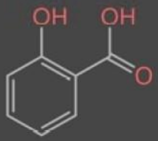	NA	220.992	0.618
DB205	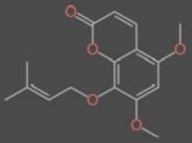	MA	4.64496	0.616
DB116	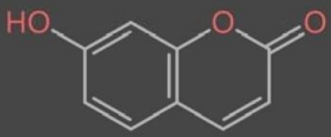	NA	32.428	0.603
DB106	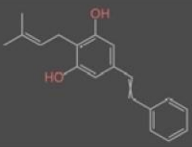	MA	3.5045	0.597
DB183	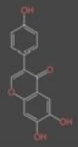	MA	17.29536	0.590
DB160	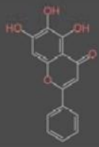	NA	34.59072	0.590
DB202	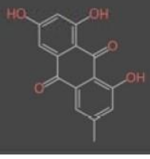	SA	1.08096	0.583

ID	Structure of Name of compounds	Class of activity	MIC(□M)	QED
DB139	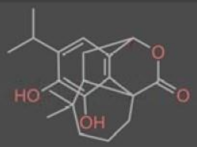	MA	5.28672	0.571
DB177	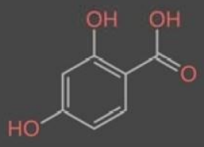	SA	0.07706	0.568
DB152	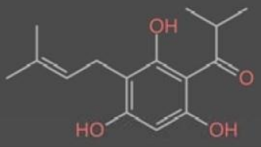	SA	2.061696	0.568
DB142	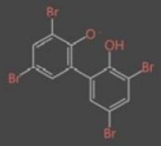	SA	0.50078	0.564
DB143	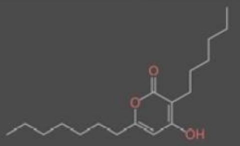	SA	1.17772	0.561
DB133	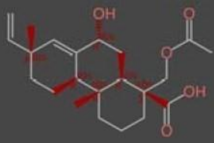	MA	6.02384	0.561
DB112	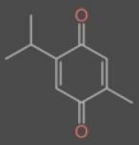	MA	2.6272	0.557
DB134	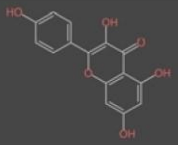	MA	3.578	0.547
DB200	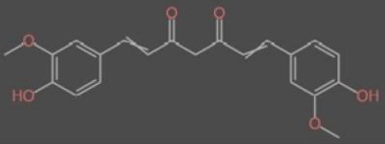	NA	92.095	0.538
DB180	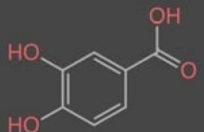	SA	0.15412	0.531
DB141	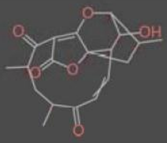	SA	1.38548	0.531
DB128	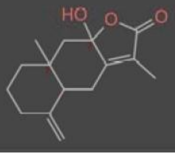	MA	7.94624	0.527

ID	Structure of Name of compounds	Class of activity	MIC(□M)	QED
DB163	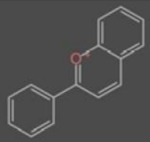	SA	0.3316	0.516
DB208	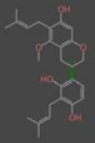	MA	3.39624	0.516
DB192	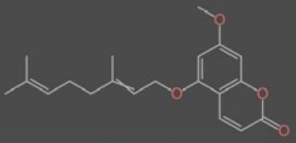	MA	5.2544	0.514
DB114	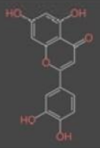	MA	3.578	0.512
DB101	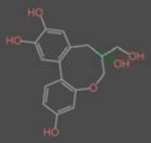	NA	38.94912	0.512
DB151	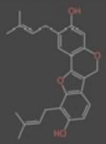	SA	0.3045666	0.507
DB186	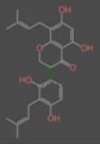	MA	3.39592	0.487
DB203	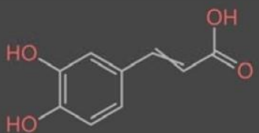	SA	0.18016	0.477
DB164	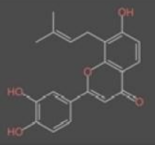	MA	5.4136	0.476
DB210	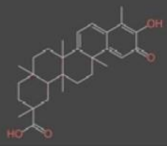	SA	0.90122	0.470
DB119	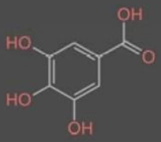	NA	51.036	0.469
DB187	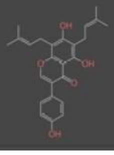	MA	3.25176	0.468

ID	Structure of Name of compounds	Class of activity	MIC(□M)	QED
DB173	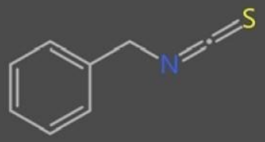	NA	29.842	0.465
DB129	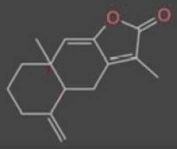	MA	14.7392	0.459
DB204	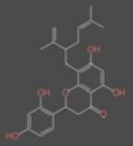	SA	1.69796	0.452
DB127	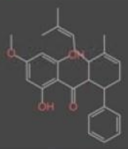	SA	0.40651	0.451
DB120	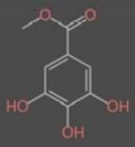	NA	46.0375	0.447
DB195	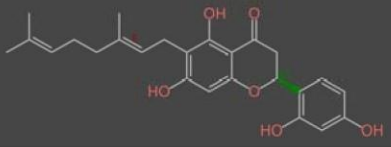	SA	0.5221227	0.446
DB154	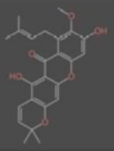	SA	1.63376	0.446
DB105	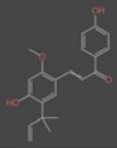	SA	2.118384	0.443
DB197	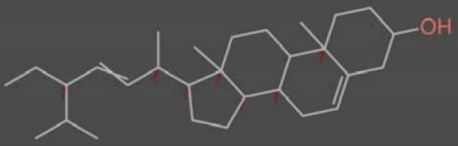	MA	2.5793125	0.443
DB137	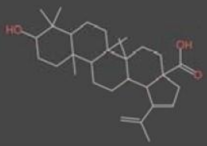	MA	14.6144	0.438
DB122	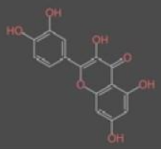	NA	151.12	0.436
DB166	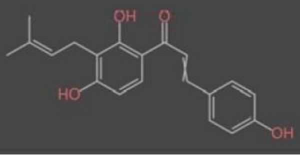	MA	2.59496	0.420

ID	Structure of Name of compounds	Class of activity	MIC(□M)	QED
DB182	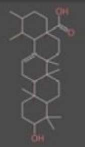	SA	1.8268	0.416
DB209	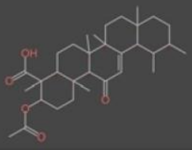	SA	1.02544	0.413
DB159	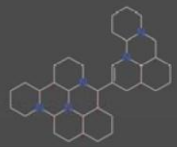	MA	3.4497088	0.411
DB124	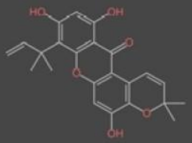	MA	6.31072	0.411
DB104	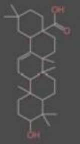	MA	7.3072	0.409
DB125	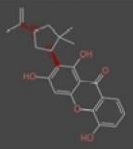	SA	1.52172	0.406
DB190	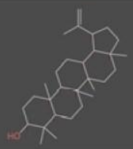	SA	1.664208	0.402
DB155	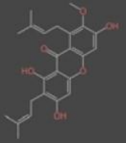	MA	3.28368	0.384
DB207	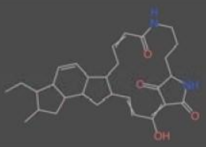	SA	0.95724	0.378
DB113	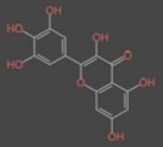	MA	19.89	0.374
DB136	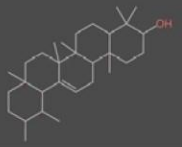	MA	13.65504	0.371
DB138	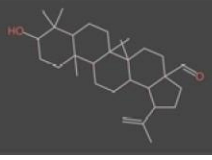	NA	112.8192	0.364

ID	Structure of Name of compounds	Class of activity	MIC(□M)	QED
DB135	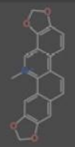	SA	1.0368696	0.358
DB115	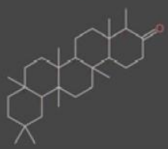	MA	4.2672	0.350
DB149	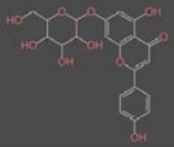	SA	1.686282	0.340
DB157	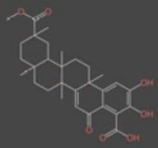	MA	5.36151	0.335
DB181	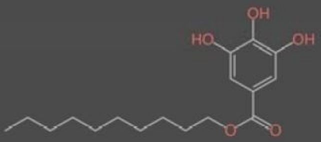	MA	3.879875	0.331
DB148	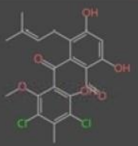	SA	0.01625373	0.319
DB126	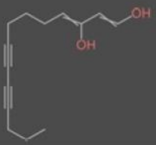	SA	1.74632	0.314
DB193	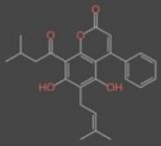	SA	0.81294	0.308
DB184	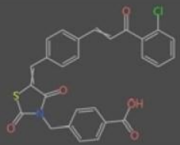	SA	0.50395	0.305
DB194	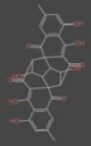	SA	0.06781125	0.288
DB167	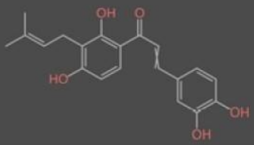	MA	5.44592	0.277
DB162	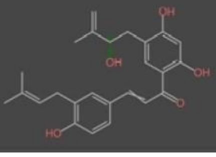	SA	0.2491789	0.276

ID	Structure of Name of compounds	Class of activity	MIC(□M)	QED
DB191	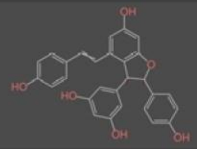	NA	45.447	0.249
DB147	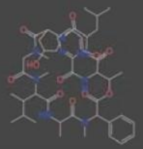	MA	14.06416	0.245
DB103	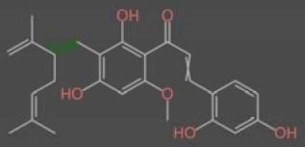	MA	3.50808	0.227
DB146	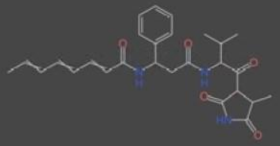	SA	0.95914	0.195
DB201	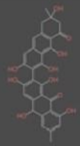	SA	2.17804	0.171
DB161	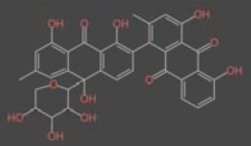	SA	1.0377558	0.139
DB171	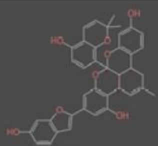	SA	0.5909498	0.131
DB211	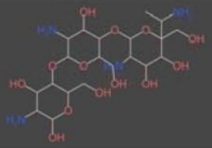	SA	0.1470285	0.109
DB169	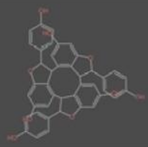	MA	6.243856	0.104
DB150	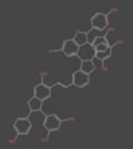	SA	1.8099	0.053
DB145	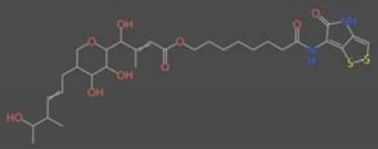	SA	0.0064081	0.052
DB175	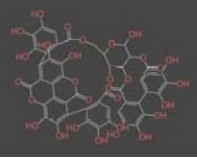	NA	66.71028	0.034

ID	Structure of Name of compounds	Class of activity	MIC(□M)	QED
DB158	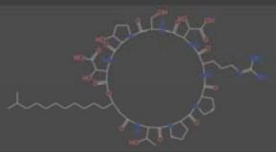	SA	1.8006612	0.022
DB156	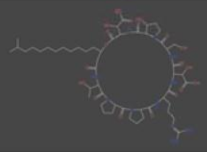	SA	0.9112662	0.021
DB170	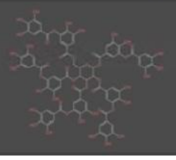	NA	435.5072	0.019

Figure S7: Drug-likeness profiling for DB169, DB211, and their analogue compared to the best scoring compound (DB123).

Molecule	Structure of SMILES	Formula	MW	cLogP	GI absorption	BBB permeant	Pgp substrate	Bioavailability Score	Synthetic Accessibility	Desirability score
DB123	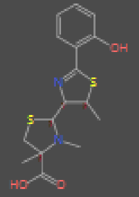	C16H20N2O3S2	352.47	1.9091	High	No	Yes	0.55	4.7	0.869
DB169	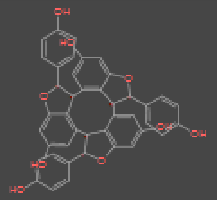	C42H30O9	678.68	5.9832	Low	No	Yes	0.17	6.41	0.143
ANA169	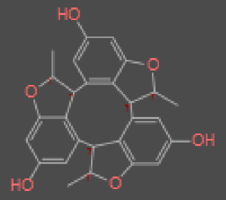	C27H24O6	444.48	3.4458	High	No	No	0.55	5.32	0.473
DB211	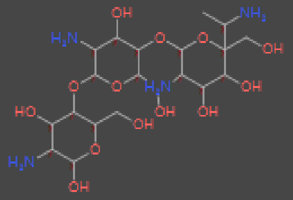	C20H40N4O13	544.55	-8.1556	Low	No	Yes	0.17	6.85	0.141
ANA211	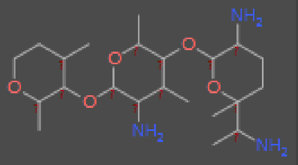	C22H43N3O5	429.59	0.1719	High	No	No	0.55	6.41	0.631

Table S2. The estimated structural-activity relationship between the AMNPs.

ID 1	ID 2	Compound 1	Compound 2	Similarity	Activity 1	Activity 2	Delta Activity	SALI
DB136	DB209	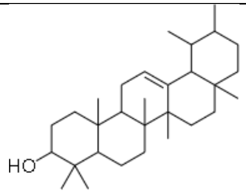	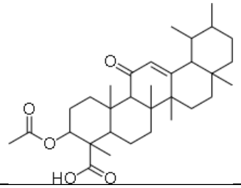	0.79893	13.65504	1.02544	12.63	62.813
DB103	DB204	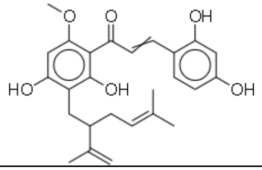	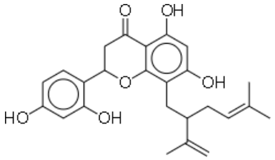	0.79944	3.50808	1.69796	1.8101	9.0252
DB195	DB204	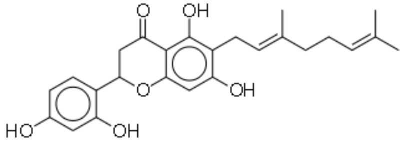	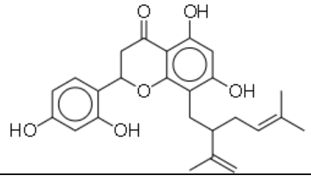	0.80031	0.5221227	1.69796	1.1758	5.8883
DB121	DB203	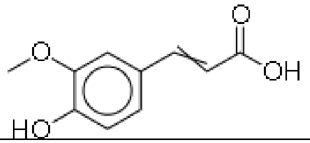	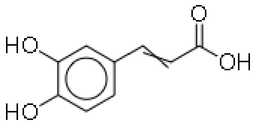	0.87695	0.09709	0.18016	0.08307	0.67508
DB137	DB190	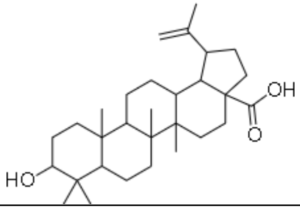	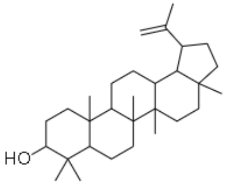	0.93422	14.6144	1.664208	12.95	196.89

DB138	DB190	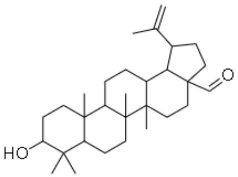	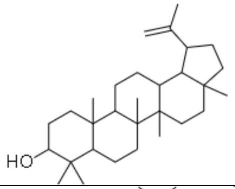	0.94215	112.8192	1.664208	111.15	1921.5
DB185	DB187	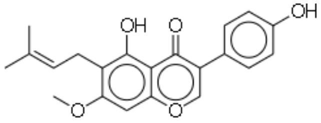	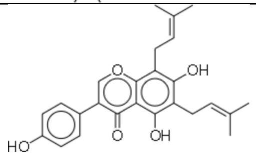	0.84147	5.63808	3.25176	2.3863	15.053
DB144	DB186	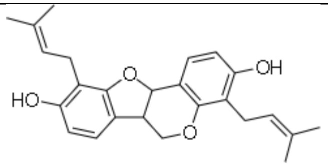	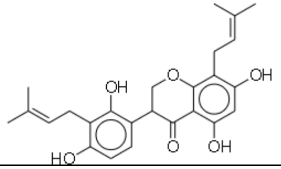	0.80879	0.3061422	3.39592	3.0898	16.159
DB155	DB186	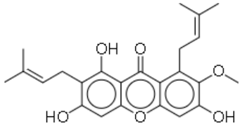	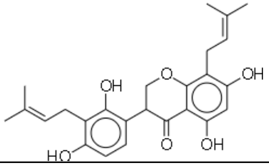	0.82079	3.28368	3.39592	0.11224	0.62632
DB165	DB185	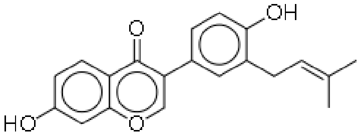	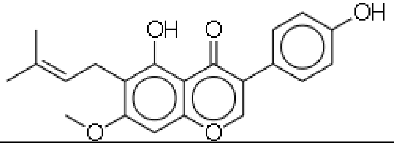	0.79747	5.1576	5.63808	0.48048	2.3723
DB104	DB182	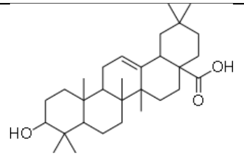	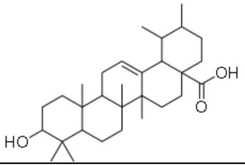	0.90226	7.3072	1.8268	5.4804	56.074
DB136	DB182	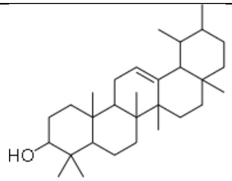	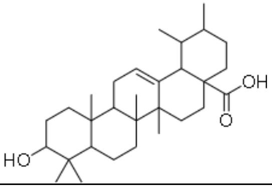	0.93422	13.65504	1.8268	11.828	179.83

DB176	DB180	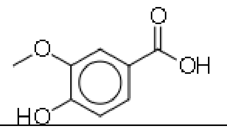	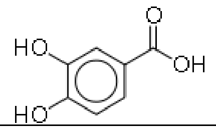	0.83876	0.084075	0.15412	0.070045	0.43441
DB150	DB169	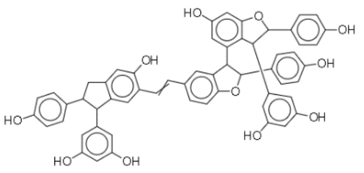	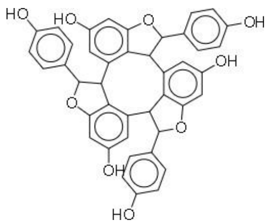	0.82016	1.8099	6.243856	4.434	24.655
DB144	DB208	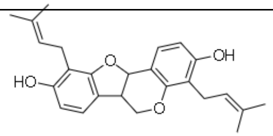	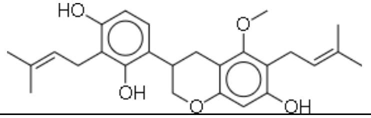	0.80879	0.3061422	3.39624	3.0901	16.161
DB155	DB208	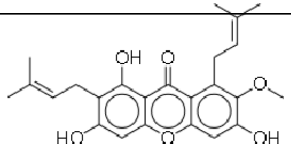	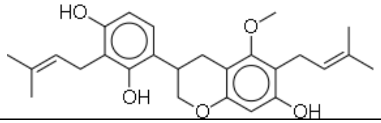	0.81103	3.28368	3.39624	0.11256	0.59564
DB186	DB208	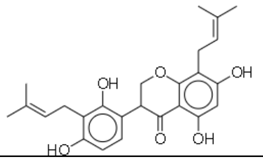	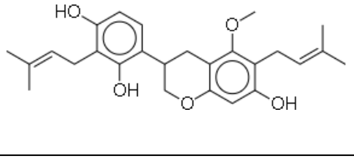	0.86981	3.39592	3.39624	3.1996e-4	0.0024576
DB164	DB167	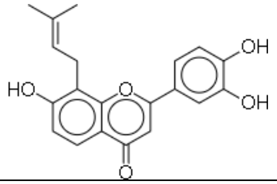	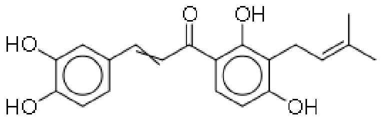	0.85658	5.4136	5.44592	0.03232	0.22535
DB166	DB167	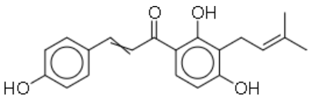	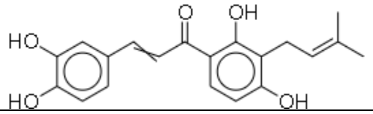	0.91204	2.59496	5.44592	2.851	32.41

DB156	DB158	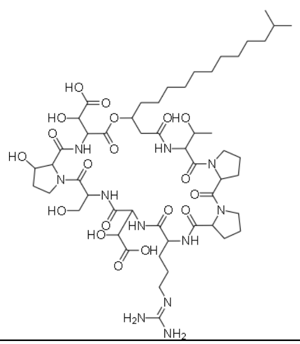	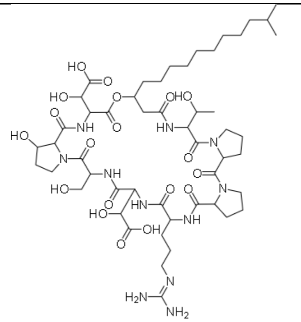	0.9991	0.9112662	1.8006612	0.88939	992.19
DB154	DB155	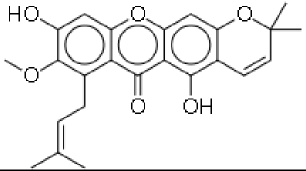	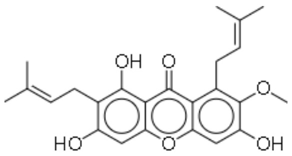	0.87063	1.63376	3.28368	1.6499	12.753
DB137	DB138	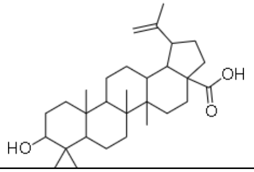	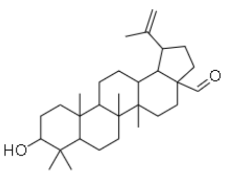	0.93165	14.6144	112.8192	98.205	1436.8
DB104	DB136	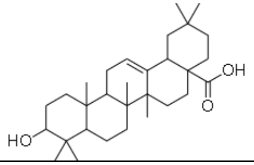	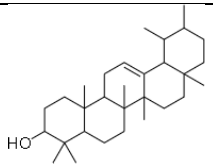	0.84799	7.3072	13.65504	6.3478	41.759
DB113	DB134	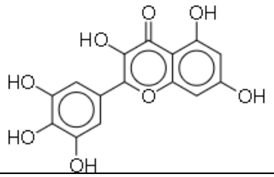	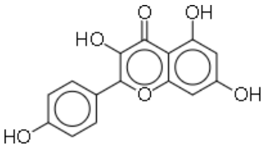	0.84052	19.89	3.578	16.312	102.28
DB122	DB134	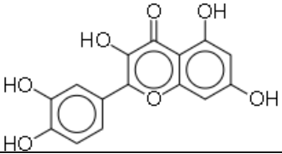	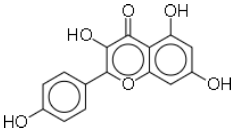	0.89145	151.12	3.578	147.54	1359.2

DB113	DB122	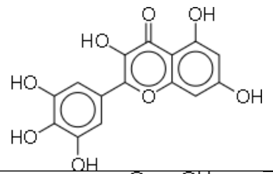	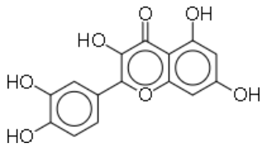	0.91768	19.89	151.12	131.23	1594.2
DB114	DB122	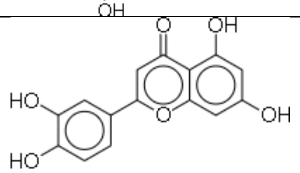	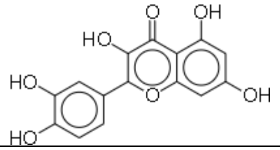	0.8696	3.578	151.12	147.54	1131.4
DB119	DB120	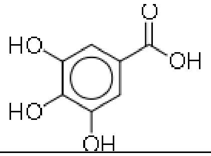	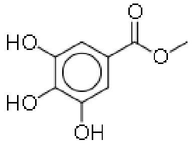	0.84884	51.036	46.0375	4.9985	33.068
DB110	DB111	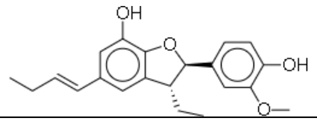	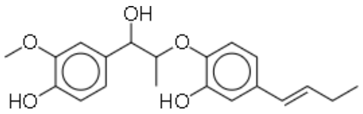	0.80547	21.78624	2.7552	19.031	97.83
DB107	DB108	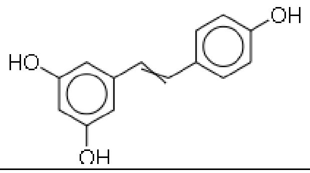	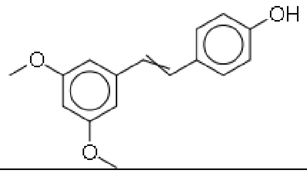	0.82862	0.2853	0.0199914	0.26531	1.5481
DB101	DB102	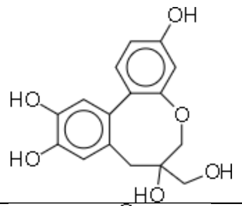	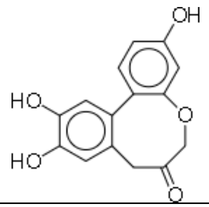	0.84495	38.94912	17.424	21.525	138.83
DB130	DB139	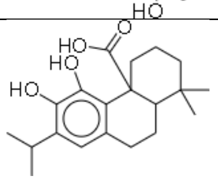	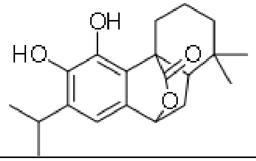	0.849	5.31888	5.28672	0.03216	0.21298

References

1. Zuo G, Han Z, Han J, Hao X, Tang H, Wang G. Antimicrobial activity and synergy of antibiotics with two biphenyl compounds , protosappanins A and B from Sappan Lignum against methicillin-resistant Staphylococcus aureus strains. J Pharm Pharmacol. 2015;67(10):1439–47.
2. Chan, B.C.L., Yu, H., Wong, C.W., Lui, S.L., Jolivalt, C., Ganem-Elbaz, C., Paris, J.M., Morleo, B., Litaudon, M., Bik-San Lau, C. and Ip, M. Quick identification of kuraridin , a noncytotoxic anti-MRSA (methicillin-resistant Staphylococcus aureus) agent from Sophora flavescens using high-speed counter-current chromatography. J Chromatogr B. 2012;880:157–62.
3. Kim S, Kim MJ, Jin D, Park S, Cho E. Antimicrobial Effect of Ursolic Acid and Oleanolic Acid against Methicillin-Resistant Staphylococcus aureus Ursolic Acid 와 Oleanolic Acid 의 메티실린 저항성 Staphylococcus aureus 에 대한 항균작용. Korean J Microbiol. 2012;48(3):212–5.
4. Cushnie TPT, Lamb AJ. International Journal of Antimicrobial Agents Recent advances in understanding the antibacterial properties of flavonoids. 2011;38:99–107.
5. Sahu NK, Balbhadra SS, Choudhary J, Kohli D V. Exploring Pharmacological Significance of Chalcone Scaffold : A Review. Curr Med Chem. 2012;19(2):pp.209-225.
6. Bruijn WJC De, Araya-cloutier C, Bijlsma J, Swart A De, Sanders MG, Waard P De, et al. Phytochemistry Letters Antibacterial prenylated stilbenoids from peanut (Arachis hypogaea). Phytochem Lett. 2018;28:13–8.
7. Yang S, Tseng C, Wang P, Lu P, Weng Y. Pterostilbene , a Methoxylated Resveratrol Derivative , Efficiently Eradicates Planktonic , Biofilm , and Intracellular MRSA by Topical Application. Front Microbiol. 2017;8:p.1103.
8. Rattanaburi S, Mahabusarakam W, Phongpaichit S, Carroll AR. Phytochemistry Letters Neolignans from Callistemon lanceolatus. Phytochem Lett. 2012;5(1):18–21.
9. Hariharan P, Gnanamani A. In vitro profiling of antimethicillin-resistant Staphylococcus aureus activity of thymoquinone against selected type and clinical strains. Lett Appl Microbiol. 2016;62(3):pp.283-289.
10. Ke S, Essid R, Mkadmini K, Ke A, Mahjoub F, Tabbene O, et al. Microbial Pathogenesis Phytochemical investigation and biological activities of Echium arenarium (Guss) extracts. Microb Pathog. 2018;118:202–10.
11. Su Y, Ma L, Wen Y, Wang H, Zhang S. Studies of the in Vitro Antibacterial Activities of Several Polyphenols against Clinical Isolates of Methicillin-Resistant Staphylococcus aureus. Molecules. 2014;19(8):12630–9.
12. Odeh IC, Tor-anyiin TA, Igoli JO, Anyam JV. In vitro antimicrobial properties of friedelan-3-one from Pterocarpus santalinoides L ' Herit , ex Dc. African J Biotechnol. 2016;15(14):531–8.
13. Monte J, Abreu AC, Borges A, Simões LC, Simões M. Antimicrobial Activity of Selected Phytochemicals against Escherichia coli and Staphylococcus aureus and Their Biofilms. Pathogens. 2014;3(2):pp.473-498.
14. Chew YL, Mahadi AM, Wong KM, Goh JK. Anti-methicillin-resistance Staphylococcus aureus (MRSA) compounds from Bauhinia kockiana Korth . And their mechanism of antibacterial activity. BMC Complement Altern Med. 2018;18(1):1–9.
15. Alves MJ, Ferreira ICFR, Froufe HJC, Abreu RM V, Martins A, Pintado M. Antimicrobial activity of phenolic compounds identified in wild mushrooms, SAR analysis and docking studies. J Appl Microbiol. 2013;115(2):346–57.
16. Júnior SD da C, Santos JV de O, Campos LA de A, Pereira MA, Magalhães NSS, Cavalcanti IMF. Antibacterial and antibiofilm activities of quercetin against clinical isolates of Staphyloccocus aureus and Staphylococcus saprophyticus with resistance profile. Int J Environ Agric Biotechnol. 2018;3(5):1948–58.
17. Oucmdz- S. Antibiotic Metabolites from the Coral-Associated Actinomycete. Chinese J Chem. 2013;31(1):100–4.
18. Thepthong P, Phongpaichit S, Carroll AR. Phytochemistry Letters Prenylated xanthones from the stem bark of Garcinia dulcis. Phytochem Lett. 2017;21:32–7.
19. Jeong S, Kim S, Kim S, Hwang B, Kwon T, Yu K, et al. Antibacterial Activity of Phytochemicals Isolated from. Molecules. 2010;15:7395–402.
20. Rukayadi Y, Lee K, Han S, Yong D, Hwang J. In Vitro Activities of Panduratin A against Clinical Staphylococcus Strains □. Antimicrob Agents Chemother. 2009;53(10):4529–32.
21. Vázquez NM, Fiorilli G, Cáceres PA, Moreno S. Phytomedicine Carnosic acid acts synergistically with gentamicin in killing methicillin-resistant Staphylococcus aureus clinical isolates. Phytomedicine. 2016;23(12):1337–43.
22. Tóth, B., Liktor-Busa, E., Urbán, E., Csorba, A., Jakab, G., Hohmann, J. and Vasas A. Fitoterapia Antibacterial screening of Juncaceae species native to the Carpathian Basin against resistant strains and LC-MS investigation of phenanthrenes responsible for the effect. Fitoterapia. 2016;115:69–73.
23. Siridechakorn I, Cheenpracha S, Ritthiwigrom T. Phytochemistry Letters Isopimarane diterpenes and flavan derivatives from the twigs of Caesalpinia furfuracea. Phytochem Lett. 2014;7:186–9.
24. Tajuddeen, N., Sani Sallau, M., Muhammad Musa, A., James Habila, D. and Muhammad Yahaya S. Flavonoids with antimicrobial activity from the stem bark of Commiphora pedunculata (Kotschy &. Nat Prod Res. 2014;28(21):pp.1915-1918.
25. Obiang-Obounou BW, Kang O-H, Choi J-G, Keum J-H, Kim S-B, Mun S-H, et al. The mechanism of action of sanguinarine against methicillin-resistant Staphylococcus aureus. J Toxicol Sci. 2011;36(3):277–83.
26. Yin P, Yong L, Navaratnam P. Fitoterapia Potential targets by pentacyclic triterpenoids from Callicarpa farinosa against methicillin-resistant and sensitive Staphylococcus aureus. Fitoterapia. 2014;94:48–54.
27. Ramírez Rueda RY. Natural Plant Products Used against Methicillin-Resistant Staphylococcus aureus. Fighting Multidrug Resistance with Herbal Extracts, Essential Oils and their Components. Academic Press; 2013. 11–22 p.
28. Zhao, J., Li, Y., Liu, Q. and Gao K. Antimicrobial activities of some thymol derivatives from the roots of Inula hupehensis. Food Chem. 2010;120(2):512–6.
29. Eom S, Kim Y, Kim S. Marine bacteria : potential sources for compounds to overcome antibiotic resistance. Appl Microbiol Biotechnol. 2013;97(11):4763–73.
30. Saleem M, Nazir M, Ali S, Hussain H, Lee S. Antimicrobial natural products : an update on future antibiotic drug candidates. Nat Prod Rep. 2010;27(2):238–54.
31. Sun, P., Maloney, K.N., Nam, S.J., Haste, N.M., Raju, R., Aalbersberg, W., Jensen, P.R., Nizet, V., Hensler, M.E. and Fenical

W. Fijimycins A-C, three antibacterial etamycin-class depsipeptides from a marine-derived Streptomyces sp. Bioorganic Med Chem. 2011;19(22):6557–62.
32. Leo L De, Lo MR, Moujir LÃ. Antibacterial properties of zeylasterone , a triterpenoid isolated from Maytenus blepharodes , against Staphylococcus aureus. Microbiol Res. 2010;165(8):pp.617-626.
33. Catteau L. Natural and hemi-synthetic pentacyclic triterpenes as antimicrobials and resistance modifying agents against Staphylococcus aureus : a review. Phytochem Rev. 2018;17(5):1129–63.
34. Cao MM, Huang SD, Di YT, Yuan CM, Zuo GY, Gu YC, et al. Myrifabine, the first dimeric myrioneuron alkaloid from myrioneuron faberi. Org Lett. 2014;16(2):528–31.
35. Guo J, Su X, Ma T, Liu J. Recent Advances on anti-MRSA Natural Products Derived from Plants. Med Res Med Res. 2018;6(10):pp.1439-1447.
36. Madanakumar AJ, Lawarence B, Manoj GS, Kumaraswamy M. Purified Anthocyanin from in vitro Culture of Bridelia retusa (L.) Spreng. capable of Inhibiting the Growth of Human Oral Squamous Cell Carcinoma Cells. Pharmacogn J. 2018;10(3).
37. Cui Y, Taniguchi S, Kuroda T, Hatano T. Constituents of Psoralea corylifolia Fruits and Their Effects on Methicillin-Resistant Staphylococcus aureus. Molecules. 2016;20(7):pp.12500-12511.
38. Seo H, Kim M, Kim S, Islam I, Nam K, Cho M, et al. Pulmonary Pharmacology & Therapeutics In vitro activity of alpha-viniferin isolated from the roots of Carex humilis against Mycobacterium tuberculosis. Pulm Pharmacol Ther. 2017;46:41–7.
39. Myint B, Sing C. Tannic acid as Phytochemical Potentiator for Antibiotic Resistance Adaptation. APCBEE Procedia. 2013;7:175–81.
40. Al-ani I, Zimmermann S, Reichling J, Wink M. Phytomedicine Pharmacological synergism of bee venom and melittin with antibiotics and plant secondary metabolites against multi-drug resistant microbial pathogens. Phytomedicine. 2015;22(2):245–55.
41. Parashar, A., Gupta, C., Gupta, S.K. and Kumar, A. Antimicrobial ellagitannin from pomegranate (Punica granatum) fruits. Int J Fruit Sci. 2009;9((3),):pp.226-231.
42. Hummelova J, Rondevaldova J, Balastikova A, Lapcik O, Kokoska L. The relationship between structure and in vitro antibacterial activity of selected isoflavones and their metabolites with special focus on antistaphylococcal effect of demethyltexasin. Lett Appl Microbiol. 2014;60:242–7.
43. Liu X, Zheng C, Sun L, Liu X, Piao H. European Journal of Medicinal Chemistry Synthesis of new chalcone derivatives bearing 2 , 4-thiazolidinedione and benzoic acid moieties as potential anti-bacterial agents. Eur J Med Chem. 2011;46(8):3469–73.
44. Dong L-M, Huang L-L, Dai H, Xu Q-L, Ouyang J-K, Jia X-C, et al. Anti-MRSA sesquiterpenes from the semi-mangrove plant Myoporum bontioides A. Gray. Mar Drugs. 2018;16(11):438.
45. El Sayed, A.M., Ezzat, S.M. and Sabry OM. A new antibacterial lupane ester from the seeds of Acokanthera oppositifolia Lam. Nat Prod Res. 2016;30(24):pp.2813.
46. Basri DF, Xian LW, Indah N, Shukor A, Latip J. Bacteriostatic Antimicrobial Combination : Antagonistic Interaction between Epsilon-Viniferin and Vancomycin against Methicillin-Resistant Staphylococcus aureus. BioMed Res Int. 2014;2014:1–9.
47. Zuo G, Wang C, Han J, Li Y, Wang G. Phytomedicine Synergism of coumarins from the Chinese drug Zanthoxylum nitidum with antibacterial agents against methicillin-resistant Staphylococcus aureus (MRSA). Phytomedicine. 2016;23(14):1814–20.
48. Mahady, G., Lawal, L.O., Raut, N. and Wick S. Natural products and traditional medicines for the treatment of multidrug resistant bacteria. Med Res Arch. 2018;6(1).
49. Radwan MM, Rodriguez-guzman R, Manly SP, Jacob M, Ross SA. Phytochemistry Letters Sepicanin A — A new geranyl flavanone from Artocarpus sepicanus with activity against methicillin-resistant Staphylococcus aureus (MRSA). Phytochem Lett. 2009;2(4):141–3.
50. Cha J, Lee J, Choi KM, Choi S, Park JH. Synergistic Effect between Cryptotanshinone and Antibiotics against Clinic Methicillin and Vancomycin-Resistant Staphylococcus aureus. Evidence-Based Complement Altern Med. 2014;2014:1–6.
51. Mailafiya, M.M., Yusuf, A.J., Abdullahi, M.I., Aleku, G.A., Ibrahim, I.A., Yahaya, M., Abubakar, H., Sanusi, A., Adamu, H.W. and Alebiosu CO. Antimicrobial activity of stigmasterol from the stem bark of Neocarya macrophylla. J Med Plants Econ Dev. 2018;2(1):1–5.
52. de Souza Pereira, J.J., Pereira, A.D.P., Jandú, J.J., da Paz, J.A., Crovella, S., dos Santos Correia, M.T. and de Azevêdo Silva J. Commiphora leptophloeos Phytochemical and Antimicrobial Characterization. Front Microbiol. 2017;8(52):1–10.
53. Teow S, Liew K, Ali SA, Khoo AS, Peh S. Antibacterial Action of Curcumin against Staphylococcus aureus : A Brief Review. J Trop Med. 2016;2016:1–10.
54. Wray V, Polavarapu PL, Pretsch A, Lin W, Kurta T. Atropisomeric Dihydroanthracenones as Inhibitors of Multiresistant Staphylococcus aureus. J Med Chem. 2013;56(8):3257–72.
55. Alves MJ, Ferreira ICFR, Froufe HJC, Abreu RM V, Martins A, Pintado M. Antimicrobial activity of phenolic compounds identified in wild mushrooms , SAR analysis and docking studies. J Appl Microbiol. 2013;115(2):346-357.
56. Lacret, R., Oves-Costales, D., Gómez, C., Díaz, C., De la Cruz, M., Pérez-Victoria, I., Vicente, F., Genilloud, O. and Reyes F. New ikarugamycin derivatives with antifungal and antibacterial properties from Streptomyces zhaozhouensis. Mar Drugs. 2015;13:128–40.
57. Raja, A.F., Ali, F., Khan, I.A., Shawl, A.S., Arora, D.S., Shah, B.A. and Taneja SC. Antistaphylococcal and biofilm inhibitory activities of acetyl-11-keto- b -boswellic acid from Boswellia serrata. BMC Microbiol. 2011;11(1):1–9.
58. Woo S, Lee S, Lee S, Lim K, Ha E, Eom Y. Activity of novel inhibitors of Staphylococcus aureus biofilms. Folia Microbiol (Praha). 2017;62(2):157–67.
59. Lee D, Kim Y, Lee M, Ahn C, Jung W, Je J. Synergistic effects between aminoethyl-chitosans and b -lactams against methicillin-resistant Staphylococcus aureus (MRSA). Bioorg Med Chem Lett [Internet]. 2010;20(3):975–8. Available from:

http://dx.doi.org/10.1016/j.bmcl.2009.12.049
